# Evaluating a multifaceted implementation strategy and package of evidence-based interventions based on WHO PEN for people living with HIV and cardiometabolic conditions in Lusaka, Zambia: protocol for the TASKPEN hybrid effectiveness-implementation stepped wedge cluster randomized trial

**DOI:** 10.1186/s43058-024-00601-z

**Published:** 2024-06-06

**Authors:** Michael E. Herce, Samuel Bosomprah, Felix Masiye, Oliver Mweemba, Jessie K. Edwards, Chomba Mandyata, Mmamulatelo Siame, Chilambwe Mwila, Tulani Matenga, Christiana Frimpong, Anchindika Mugala, Peter Mbewe, Perfect Shankalala, Pendasambo Sichone, Blessings Kasenge, Luanaledi Chunga, Rupert Adams, Brian Banda, Daniel Mwamba, Namwinga Nachalwe, Mansi Agarwal, Makeda J. Williams, Veronica Tonwe, Jake M. Pry, Maurice Musheke, Michael Vinikoor, Wilbroad Mutale

**Affiliations:** 1 Centre for Infectious Disease Research in Zambia (CIDRZ), Lusaka, Zambia; 2 Institute for Global Health and Infectious Diseases, University of North Carolina, Chapel Hill, NC USA; 3 Department of Biostatistics, School of Public Health, University of Ghana, Accra, Ghana; 4 Department of Health Economics, School of Public Health, University of Zambia, Ridgeway Campus, Lusaka, Zambia; 5 Department of Health Promotion and Education, School of Public Health, University of Zambia, Ridgeway Campus, Lusaka, Zambia; 6 Department of Epidemiology, University of North Carolina, Chapel Hill, NC USA; 7 Department of Paediatrics and Child Health, School of Medicine, University of Zambia, Lusaka, Zambia; 8 Department of Medicine, Division of Infectious Diseases, University Teaching Hospital, Lusaka, Zambia; 9 Institute of Public Health, School of Medicine, Washington University in St. Louis, St. Louis, MO USA; 10 Center for Translation Research and Implementation Science, National Heart, Lung, and Blood Institute, U.S. National Institutes of Health, Bethesda, MD USA; 11 Department of Epidemiology, School of Medicine, University of California at Davis, Davis, CA USA; 12 Division of Infectious Diseases, Department of Medicine, University of Alabama, Birmingham, AL USA; 13 Department of Health Policy and Management, School of Public Health, University of Zambia, Lusaka, Zambia

**Keywords:** Non-communicable diseases, HIV/AIDS, Hypertension, Diabetes, Dyslipidemia, Task shifting, Integration, Stepped-wedge trial, Hybrid effectiveness-implementation trial, Zambia

## Abstract

**Background:**

Despite increasing morbidity and mortality from non-communicable diseases (NCD) globally, health systems in low- and middle-income countries (LMICs) have limited capacity to address these chronic conditions, particularly in sub-Saharan Africa (SSA). There is an urgent need, therefore, to respond to NCDs in SSA, beginning by applying lessons learned from the first global response to any chronic disease—HIV—to tackle the leading cardiometabolic killers of people living with HIV (PLHIV). We have developed a feasible and acceptable package of evidence-based interventions and a multi-faceted implementation strategy, known as “TASKPEN,” that has been adapted to the Zambian setting to address hypertension, diabetes, and dyslipidemia. The TASKPEN multifaceted implementation strategy focuses on reorganizing service delivery for integrated HIV-NCD care and features task-shifting, practice facilitation, and leveraging HIV platforms for NCD care. We propose a hybrid type II effectiveness-implementation stepped-wedge cluster randomized trial to evaluate the effects of TASKPEN on clinical and implementation outcomes, including *dual control* of HIV and cardiometabolic NCDs, as well as quality of life, intervention reach, and cost-effectiveness.

**Methods:**

The trial will be conducted in 12 urban health facilities in Lusaka, Zambia over a 30-month period. Clinical outcomes will be assessed via surveys with PLHIV accessing routine HIV services, and a prospective cohort of PLHIV with cardiometabolic comorbidities nested within the larger trial. We will also collect data using mixed methods, including in-depth interviews, questionnaires, focus group discussions, and structured observations, and estimate cost-effectiveness through time-and-motion studies and other costing methods, to understand implementation outcomes according to Proctor’s Outcomes for Implementation Research, the Consolidated Framework for Implementation Research, and selected dimensions of RE-AIM.

**Discussion:**

Findings from this study will be used to make discrete, actionable, and context-specific recommendations in Zambia and the region for integrating cardiometabolic NCD care into national HIV treatment programs. While the TASKPEN study focuses on cardiometabolic NCDs in PLHIV, the multifaceted implementation strategy studied will be relevant to other NCDs and to people without HIV. It is expected that the trial will generate new insights that enable delivery of high-quality integrated HIV-NCD care, which may improve cardiovascular morbidity and viral suppression for PLHIV in SSA. This study was registered at ClinicalTrials.gov (NCT05950919).

**Supplementary Information:**

The online version contains supplementary material available at 10.1186/s43058-024-00601-z.

Contributions to the literature

1. This paper presents an evidence-based package called “TASKPEN,” based on the WHO package of essential noncommunicable disease (PEN) interventions and a multifaceted implementation strategy centered on reorganizing clinical services and task-shifting, to facilitate integration of cardiometabolic non-communicable disease (NCD) care into U.S. President’s Emergency Plan for AIDS Relief (PEPFAR)-supported HIV clinical settings in Zambia.
2. The paper introduces a practical definition for measuring control of both HIV and cardiometabolic NCDs called “dual control”.
3. The study will provide new knowledge on the: effects of the TASKPEN package on dual control; health system consequences of HIV/NCD integration; and ways to apply selected outcomes and domains of the Consolidated Framework for Implementation Research (CFIR), Proctor’s Outcomes for Implementation Research, and RE-AIM to evaluate HIV/NCD service integration in resource-constrained routine practice settings in sub-Saharan Africa.

## Introduction

Globally, non-communicable diseases (NCDs) pose a significant public health and economic threat due to the disability, loss of productivity, and premature deaths associated with these conditions [1]. Among the NCDs, cardiometabolic NCDs, including cardiovascular disease (CVD), are the leading causes of mortality, contributing to 17.9 million of the estimated 41 million NCD-related deaths globally in 2023 [2]. In sub-Saharan Africa (SSA), CVDs are on a trajectory to overtake infectious diseases, such as HIV, as the leading causes of death by the year 2030. Zambia, like many SSA countries, is experiencing an increased burden of cardiometabolic NCDs because of improving economic conditions, more sedentary lifestyles, and increasingly Western-style diets and substance use, including unhealthy alcohol and tobacco use [3–5].

This epidemiological transition disproportionately affects people living with HIV (PLHIV). With the increasing availability of antiretroviral therapy (ART), efforts to end the HIV epidemic in Africa have slowly begun to address HIV-related non-communicable co-morbidities. Hypertension afflicts 8.9 million PLHIV globally, and, while prevalence varies by region, it affects nearly 20% of all PLHIV in Eastern and Southern Africa [6, 7]. Similarly, cardiometabolic complications related to diabetes are increasing among PLHIV in Zambia and the region [8], with prevalence estimates exceeding 5% and 15% for diabetes mellitus and prediabetes, respectively [9]. Diabetes mellitus among PLHIV is associated with 2.4 times the risk of CVD events compared to HIV-uninfected persons [10]. Antiretroviral therapy (ART) regimens further complicate CVD risk, with newer antiretroviral drugs such as dolutegravir potentially associated with weight gain and blood pressure increase, and others, such as protease inhibitors, with dyslipidemia [8, 11]. Changes in body composition associated with some antiretroviral agents, including excess visceral adipose tissue, have been linked to increased mortality [12, 13].

Evidence-based tools exist for the management of NCDs in low- and middle-income country (LMIC) primary care settings. For example, the World Health Organization (WHO) has developed a *Package of Essential Noncommunicable Disease Interventions for Primary Health Care* (WHO PEN) which is a collection of evidence-based practices that includes: clinical decision support for managing cardiometabolic NCDs via easy-to-follow algorithms; a lifestyle counselling curriculum; and streamlined drug treatment protocols [14]. Its efficacy at improving blood pressure control is well proven in LMICs [15, 16]. Despite being established as an evidence-based intervention, implementation of WHO PEN and related tools is rare in SSA, and strategies to integrate it within HIV services has not been explored.

Strategies that could be relevant include re-configuring service sites to enable colocation of integrated HIV and NCD services and task shifting NCD screening and management to non-physician health workers (NPHW). Several integration approaches have been piloted for NCD screening [17, 18] and HIV care and treatment [19, 20], however, most have focused on cancer and mental health [21]. Task shifting is common and effective in HIV care in SSA, but has not yet been widely extended to other chronic conditions [22, 23]. Because the professional workforce in many SSA countries, including Zambia, remains limited, task shifting hypertension care to nurses or community health workers (CHWs) has the potential to synergize resources and knowledge to support NCD care [22, 24]. Just as non-physicians now have strong capacity to provide HIV care using a public health approach, hypertension care can be provided by NPHWs who counsel patients on the benefits of a healthy lifestyle almost as often as physicians [22, 25].

The overarching objective of this study is to evaluate a package of evidence-based practices (EBPs) based on WHO PEN, together with an associated multi-faceted implementation strategy centered around task shifting and collocating health services in HIV care settings, which we have collectively referred to as *TASKPEN* (Tables 1 and 2). TASKPEN was developed through formative research that adapted the WHO PEN training curricula and algorithms to the Zambian HIV care setting for use by nurses and other NPHWs. We now plan to test the finalized TASKPEN package in a hybrid type II effectiveness-implementation stepped-wedge trial. In this paper, we describe the trial design, aims, outcomes, procedures, and novel analytical approaches to overcome threats to validity and generalizability. By rigorously evaluating the TASKPEN package, our study examines a potentially transformative approach to managing the intersecting challenges of HIV and NCDs in Zambia—one that may significantly advance healthcare for PLHIV with comorbid cardiometabolic conditions.

**Table 1:** Overview of evidence-based practices (EBPs) and multi-faceted implementation strategy that form the TASKPEN intervention package, with supporting evidence cited

TASKPEN Evidence-based Practice (EBP) Components	Description of EBP Component	Implementation Barrier	Multi-faceted Implementation Strategy
1. **One-stop shop for HIV and NCD screening, diagnosis, treatment, and care**	A “one stop shop” enables co-management of HIV and NCDs in an Integrated ART/NCD Clinic. Integrated NCD care will take advantage of existing innovations in HIV care, including for medication distribution, differentiated service delivery (DSD), and integrated adherence support and back-to-care services	1. Siloed care: HIV and NCDs [26]	1. ***Main strategy—Change/ Integrate service sites***: Re-configure location for NCD service delivery such that TASKPEN/ integrated NCD care is available in ART and DSD HIV clinics and leverages the PEPFAR platform.^40, 41^
2. **WHO PEN guidelines and training materials**	A WHO-recommended NCD technical package has been harmonized with national guidelines adapted for non-physician health worker-led management in Zambia, with clear guidelines on when to consult or refer patients to specialist physician and higher-level facilities. Training materials are protocolized and feature an emphasis on patient-centred care. Nurses will be trained on the WHO PEN protocols and will be enabled to prescribe primary-level medications for hypertension, diabetes, and dyslipidemia. Training sessions will include pre- and post-test training of understanding and team-based interactive review of test answers. Community health workers based at the facility will conduct blood pressure screening, height and weight measurement, and random blood glucose monitoring. In addition, they will support health education, including delivery of brief medication adherence and smoking cessation counselling, and also support community tracing and back to care activities for PLHIV with cardiometabolic complications who are late for a medication collection or who disengage from care	2. Lack of adequate technical skills in NCD management [5, 27] 3. Shortage of human resources for health, particularly doctors [27]	a. Practice facilitation: Provide training, educational materials, and support to facilitate healthcare worker skills acquisition on integrated NCD management b. Revise professional roles: Task shift integrated NCD management to non-physician health workers, building on existing task shifting practices used in routine HIV care
3. **Electronic Medical Record (EMR)**	The local EMR for PLHIV in Zambia, SmartCare, has been adapted to include data collection fields and clinical decision support prompts for NPHWs to manage cardiometabolic NCDs. Like for HIV parameters, the EMR also can track progress on performance and process indicators, which will be given to providers to facilitate site-level quality improvement	4. Lack of data for clinical follow-up, decision support, and program monitoring & evaluation [27, 28]	c. Change record systems: Add NCD-focused module to national HIV EMR (known as SmartCare) [29] with integrated decision support/ automatic clinical reminders d. Audit & Feedback: Regular collaborative data review meetings among frontline health workers focused on dashboard indicators to identify and act on areas for quality improvement
4. **Cardiometabolic condition diagnostic testing and monitoring**	Point-of-care (POC) diagnostics and/or strengthened laboratory systems to enable screening and diagnostic testing for cardiometabolic NCDs at health facility level. EBP also includes decentralized monitoring of NCDs/ cardiovascular risk factors	5. Lack of basic diagnostic and vital sign equipment [30]	d. Change physical structure and equipment: Provide basic NCDs diagnostic, laboratory, and vital sign equipment
5. **Strengthened NCD medication supply chain, including multi-month dispensing (MMD)**	TASKPEN teams will engage MOH leadership to support improved forecasting and delivery of cardiometabolic NCD medications. TASKPEN practice facilitators and MOH champions based at the clinics will support facility-level logistic and supply chain management, MMD of NCD medications within the existing PEPFAR program, and emergency provision of NCD medications in case of stock outs	6. Stock outs of recommended anti-hypertensive, anti-diabetic, and anti-lipid medications	e. Use work groups: Engage MOH governing structures in enhanced NCD medication supply chain management at central levels to build support for NCD MMD in ART clinics f. Practice facilitation: Provide training, educational materials, and support to facilitate healthcare worker skills acquisition on pharmacy management and MMD implementation

**Table 2:** Description of components of the multi-faceted TASKPEN implementation strategy

**Implementation strategy **	**Main or sub-component strategy**	**Implementation actors**^a^	**Implementation action**^a^
1) Change/ Integrate service sites	**Main**	CIDRZ/ TASKPEN Practice Facilitators MOH HIV/NCD “Integration Champions”	CIDRZ/TASKPEN Practice Facilitators will work with MOH HIV/NCD Integration Champions to reorganize patient flow at study sites to better integrate HIV and NCD care. This will be done by changing the service site for NCD care for PLHIV from outpatient departments to the ART and differentiated service delivery (DSD) clinics. CIDRZ/ TASKPEN Practice Facilitators will introduce features of integrated NCD care into existing HIV service delivery platforms, building on PEPFAR and national HIV investments
a. Practice facilitation b. Revise professional roles	Sub-component	CIDRZ/ TASKPEN Practice Facilitators; MOH HIV/NCD Integration Champions	CIDRZ/TASKPEN Practice Facilitators will train and coach MOH CHWs, peers, and nurses to assume NCD care responsibilities. CIDRZ/ TASKPEN Practice Facilitators and MOH HIV/NCD Integration Champions will engage in interactive problem solving and support basic quality improvement activities that enhances integrated care delivery
c. Change record systems d. Audit & Feedback	Sub-component	CIDRZ/ TASKPEN Medical Informatics team; CIDRZ/ TASKPEN Practice Facilitators; MOH HIV/NCD Integration Champions	CIDRZ/ TASKPEN Medical informatics team will introduce and manage a new module in the HIV EMR (SmartCare) to record cardiometabolic NCD data for PLHIV. The module will provide basic decision support to MOH NPHWs. It will also facilitate a simple automated dashboard for NCD performance indicator monitoring, which will be reviewed with NPHWs by CIDRZ/ TASKPEN Mentors and MOH HIV/NCD Integration Champions during data review meetings led by MOH staff. Dashboard indicators will focus on identifying processes for quality improvement (i.e., % of PLHIV with documented blood pressure measurement in reporting period, % of PLHIV with hypertension with anti-hypertensive medication collection in reporting period, etc.)
e. Change physical structure and equipment	Sub-component	CIDRZ/ TASKPEN Practice Facilitators;	CIDRZ/ TASKPEN laboratory technologist(s) will offer practice facilitation and support to strengthen NCD laboratory systems at facility level. POC platforms may be introduced to increase screening for diabetes and dyslipidemia in selected study sites if the facility does not have an existing chemistry platform or sufficient reagents or trained lab personnel to conduct these tests at the local lab. CIDRZ/ TASKPEN Practice Facilitators will add digital sphygmomanometers and glucometers to clinical screening areas in HIV service delivery spaces
f. Use Working Groups g. Practice facilitation	Sub-component	MOH Leadership; CIDRZ/ TASKPEN Practice Facilitators; MOH HIV/NCD Integration Champions;	MOH governing structures, such as ZAMRA and ZAMMSA, will be engaged through TASKPEN working groups to forecast and procure essential NCD medications. CIDRZ/ TASKPEN working groups will provide advice on medication supply chain management to MOH management. At facility level, CIDRZ/ TASKPEN Practice Facilitators and MOH Integration Champions will notify clinicians about available NCD medications and support multi-month dispensation of anti-hypertensive, anti-diabetic, and anti-lipid medications as stock levels allow

^a^Implementation strategy specification per Proctor et al. [31]

## Methods

### Study design

**Fig. 1:**
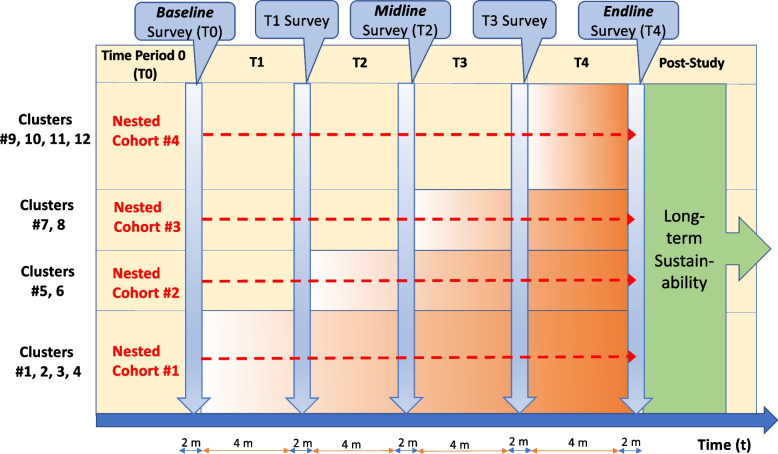
Incomplete stepped-wedge design schematic for TASKPEN evaluation. Orange blocks reflect randomly selected cluster transition to the TASKPEN intervention

We will conduct a hybrid type II effectiveness-implementation stepped-wedge cluster randomized trial, which is a pragmatic quasi-experimental design that will allow us to assess both implementation and clinical effectiveness outcomes, and thereby accelerate translation of our findings into policy and practice. With this design, we plan to achieve the following study aims: 1) estimate the effectiveness of the TASKPEN intervention on ‘dual control’ of HIV and cardiometabolic NCDs, as well as measures of cardiovascular risk and quality of life; and 2) evaluate TASKPEN implementation outcomes, including cost-effectiveness. Following a stepped-wedge approach, sequential crossover of sites (i.e., a health facility and surrounding catchment areas) will take place from standard of care (tan) to intervention (i.e., TASKPEN package shaded in orange) after a cross-sectional assessment of patient outcomes until all 12 sites (i.e., clusters) are exposed to the intervention (Fig. 1). As illustrated in the figure, outcome measurement will happen at times T0 [baseline], T1, T2 [midline], T3, and T4 [endline]. To overcome the limitations inherent to cross-sectional assessments of patient outcomes, and to facilitate granular longitudinal data collection, we will also enroll a prospective cohort at 4 randomly selected study sites (i.e., dashed red arrows), “nested” in the larger trial. The cohort will comprise a representative sample of survey participants with co-morbid cardiometabolic NCDs and risk factors identified through the surveys to longitudinally follow throughout the study.

**Fig. 2:**
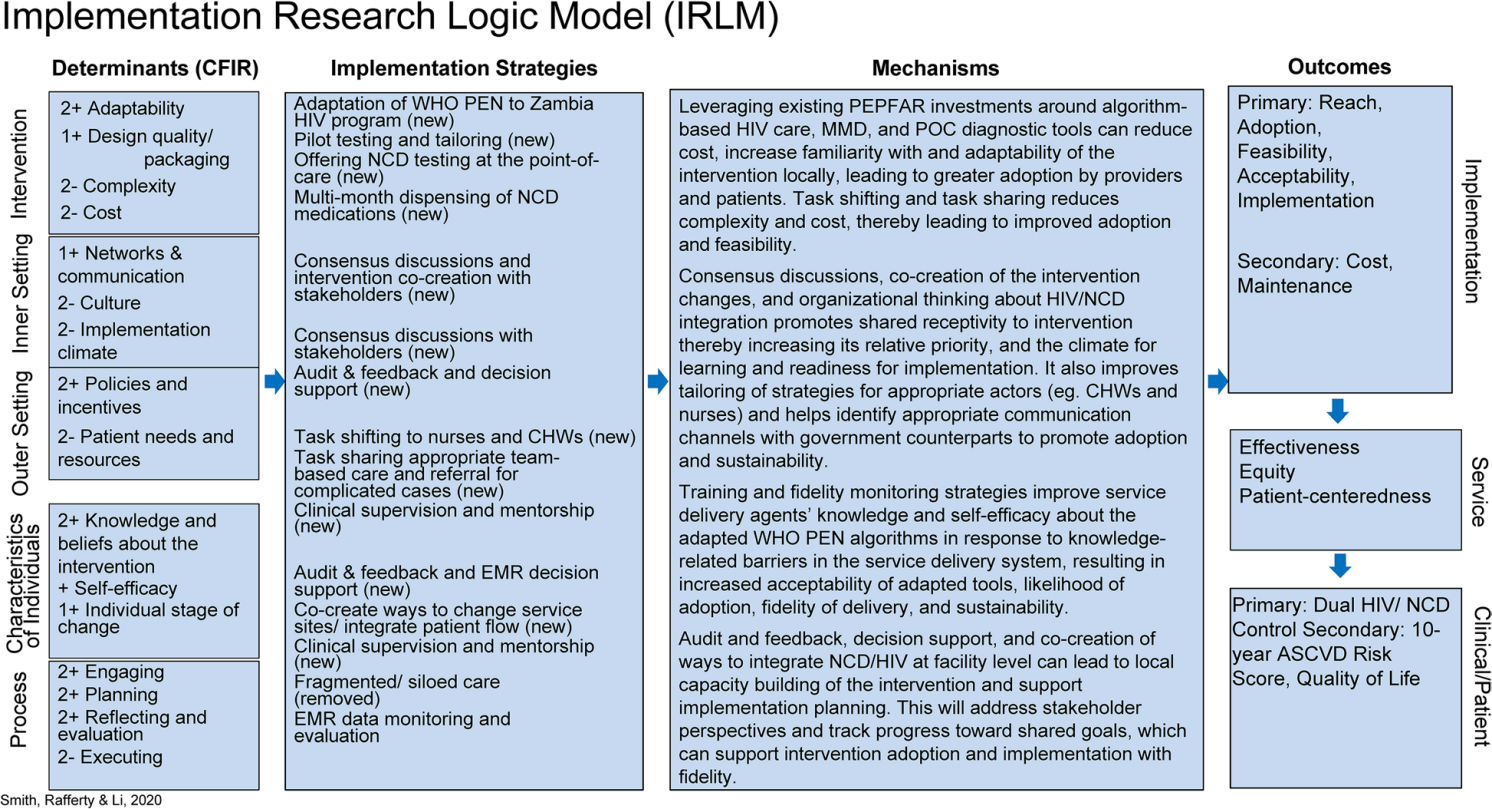
Implementation Research Logic Model adapted to the TASKPEN intervention [36]

To support introduction of the TASKPEN package in the U.S. President’s Plan for AIDS Relief (PEPFAR)-supported HIV program in Zambia, we have borrowed from the Consolidated Framework for Implementation Research (CFIR) [32, 33] and RE-AIM (Reach, Evaluation, Adoption, Implementation and Maintenance) [34, 35]. Using a project-specific conceptual model and the Implementation Research Logic Model (Fig. 2), we delineate the complex relationships between barriers to introducing the TASKPEN package and the underlying mechanisms that lead to outcomes of interest. These frameworks organize our thinking about how the TASKPEN package may act on different barriers to achieve implementation outcomes, which, in turn, have effects on important service and patient outcomes, including quality of life and ‘dual control’ of HIV and cardiometabolic NCDs for PLHIV.

### Study setting and population

Twelve high-volume sites (i.e., each site has ≥ 3,000 PLHIV in care per most recent PEPFAR data) were selected to participate in the trial from among all public health facilities in Lusaka, Zambia. The study sites reflect different levels of the Zambian health system. Prior to trial launch, we will review our formative site assessment data and equip all sites with basic NCD equipment at the clinical departments where most PLHIV seek and receive care and treatment (i.e., ART and differentiated service delivery [DSD] clinics) to ensure a basic standard of care (SOC) at baseline.

The target study population is all adult PLHIV accessing HIV care and treatment services in Lusaka, Zambia. For patient surveys, all PLHIV ≥ 18 years enrolled in HIV care at the study sites during the study period will be eligible for inclusion. For the nested cohort, eligible participants will need to have completed a study survey and have ≥ 1 of the following cardiometabolic conditions or risk factors:

- 1) Any current tobacco use (within 30 days of the survey, whether daily or non-daily);
- 2) Hypertension as defined by WHO PEN/ HEARTS (i.e., systolic blood pressure [SBP] ≥140 mmHg and/or diastolic blood pressure [DBP] ≥90 mmHg); [37]
- 3) Diabetes mellitus as defined by WHO PEN/ HEARTS (i.e., random plasma glucose ≥ 11.1 mmol/L, fasting plasma glucose ≥ 7 mmol/L, and/or hemoglobin A1c ≥ 48 mmol/mol or ≥6.5%; and/or compatible clinical diagnosis); [37]
- 4) Prediabetes (defined as having impaired fasting glucose of 6.1–6.9 mmol/L and/or hemoglobin A1c 42–48 mmol/mol or between 6.0–6.4%); and/or
- 5) Dyslipidemia (defined as total cholesterol ≥5.2 mmol/L or low-density lipoprotein ≥3.4 mmol/L).

### Trial standard of care

While screening, diagnosis, and treatment of cardiometabolic NCDs for PLHIV are recommended by national HIV guidelines, these services are generally unavailable or inconsistent in most ART and DSD clinics leading to low rates of NCD control [5, 38]. Equipment for blood pressure measurement is often available in ART and DSD clinics, but hypertension screening is not normalized nor universal in such clinics. Most public health facilities do not offer fasting or random blood glucose screening or hemoglobin A1c and lipid panel testing [5]. Fragmented NCD supply chain management systems also mean that essential medications for hypertension, diabetes, and dyslipidemia are often unavailable, and, when in stock, often can only be dispensed one to a few weeks at a time, thereby leading to frequent treatment interruption. Across facilities, health worker capacity to diagnose and manage cardiometabolic NCDs is limited and not protocolized like HIV services. Under the SOC, NPHWs such as clinical officers oversee clinical service delivery in virtually all ART and DSD clinics and prescribe ART and treatment for opportunistic infections.

### Trial intervention

TASKPEN is a package of five EBP components that enables introduction of WHO PEN via a multi-faceted implementation strategy centred on task-shifting and service colocation for integration within routine PEPFAR-supported HIV care settings in Zambia. TASKPEN is designed to build upon and strengthen the SOC. The EBP components and multi-faceted implementation strategy are described in detail in Tables 1 and 2.

### Study outcomes

Table 3 describes primary and secondary clinical effectiveness outcomes for the trial. Our primary clinical outcome, ‘dual control,’ is defined as HIV viral suppression *and* control of all comorbid cardiometabolic NCDs and associated risk factors—namely, hypertension, diabetes, dyslipidemia, and smoking—according to established criteria [39, 40]. Table 4 describes study implementation outcomes.

**Table 3:** Primary and secondary clinical effectiveness outcomes of interest

Primary outcome	**Definition (& Time Points)**	**Notes**
*Dual* HIV and NCD Control	# and % of participants with the following at **12 months**: 1. HIV RNA < 1,000 copies (c)/ml on most recent measure **AND** 2. Absence of the following: 1) uncontrolled systolic and diastolic hypertension; 2) uncontrolled diabetes mellitus; and 3) current (past 30 days) tobacco smoking	*Uncontrolled* systolic hypertension implies measure ≥ 140 mmHg; *uncontrolled* diastolic hypertension implies measure ≥ 90 mmHg; *Uncontrolled* diabetes mellitus implies Hgb A1c ≥ 8% (or most recent fasting blood glucose > 7 mmol/L)
Secondary Outcomes	**Definition (& Time Points)**	**Notes**
10-year ASCVD Risk	# and % of participants who: Experience numerical improvement in 10-year ASCVD risk score at 12 and 24 months Experience improvement in risk category at 12 and 24 months	Risk scores and categories according to the American College of Cardiology ASCVD Pooled Cohort Risk Estimator (https://tools.acc.org/ascvd-risk-estimator-plus/#!/calculate/estimate/):
Blood Pressure Control	Average numerical change in systolic and diastolic blood pressure from baseline at 12 and 24 months	Among survey participants presenting for ART/ HIV care services at study sites, as well as within individuals enrolled in the nested cohort
Severe Hypertension	# and % of participants with severe hypertension at 0, 12, and 24 months	Defined as systolic blood pressure ≥ 180 mmHg and/or diastolic blood pressure ≥ 120 mmHg
Diabetes Control	Average change in hemoglobin A1c and fasting glucose from baseline at 12 and 24 months	Among survey participants presenting for ART/ HIV care services at study sites, as well as within individuals enrolled in the nested cohort; mean change in HgbA1c and/or fasting blood glucose from baseline at last assessment
Dyslipidemia Control	Average change in LDL and/or total cholesterol from baseline at 12 and 24 months	Mean change in total cholesterol and LDL from baseline at last assessment
Ideal Cardiovascular Health (CVH)	Average change in Ideal CVH score on a 0–7 point Likert scale at times 0, 12, and 24 months	Change in 7-point scale defined according to the American Heart Association Life Simple 7 Factors (https://www.ahajournals.org/doi/10.1161/circulationaha.109.192703)
HIV-1 Viral Suppression	# and % of participants with HIV-1 RNA viral suppression at 0, 12, and 24 months	Assessed at empirically supported thresholds of < 1,000 c/ml, < 200 c/ml, and < 50 c/ml
Retention in HIV Care	# and % of participants with evidence of being retained in HIV care within the last ~ 180 days at 0, 12, and 24 months	Participants without evidence of HIV treatment interruption, death, or loss to follow up
Medication adherence	Variation of medication possession ratio (MPR)	The number of days late for pharmacy refills by total days on treatment for ART and NCD medications at 0, 12, and 24 months
Quality of Life	# and % of participants with an increase in quality-of-life score at 12 and 24 months	Quality of life score assessed by WHOQOL-HIV BREF for Zambia: doi:10.1016/j.jana.2010.04.006

**Table 4:** Implementation outcomes of interest

Implementation Outcome	Definition	Theoretical Basis	Measure(s)
Reach	**No. of people and percentage of the target population affected and the extent to which the individuals reached are representative and include those most at risk**	RE-AIM	• *# and % of PLHIV at the facility (i.e., with documented receipt of ART/ HIV care services at a study site) screened for hypertension at 0, 12, and 24 months from TASKPEN introduction*
Adoption	**No. and % of settings participating, and the extent to which the settings selected are representative of settings that the target population will use or visit**	RE-AIM, CFIR	• *% of facilities and providers initiating TASKPEN intervention/ integrated care at 0, 12, and 24 months* • *Perceived enablers and barriers to TASKPEN implementation and perceived fit with organizational priorities and work flows as assessed through interviews and focus groups at* ≥ *6 months from TASKPEN introduction*
Implementation	**Level of adherence to implementation principles or guidelines, intervention’s protocol, including cost and consistency of delivery as intended**	RE-AIM, CFIR	• *% of nurses and other non-physician health workers at each site that supported intervention/ integrated care implementation at least once at 0, 12 and 24 months* • *Fidelity to TASKPEN guidelines and training materials/ SOPs as assessed by observer assessment during structured observations pre- and post-TASKPEN intervention at each site at* ≥ *6 months from TASKPEN introduction* • *Site-level adaptations to TASKPEN delivery and its management at different clinics as assessed by structured observations, FGDs, and KIIs done* ≥ *6 months from TASKPEN introduction* • *Total costs for TASKPEN implementation*
Acceptability	Extent to which implementation stakeholders perceive a treatment, service, practice, or innovation to be agreeable, palatable, or satisfactory	RE-AIM, CFIR	• *Average (mean) Acceptability of Intervention Measure (AIM) score after TASKPEN implementation at* ≥ *6 months from TASKPEN introduction* • *Perceptions at* ≥ *6 months from TASKPEN introduction among interview and FGD participants regarding the extent to which TASKPEN is agreeable/ desirable to them*
Fidelity	Participants with evidence of NCD treatment interruption/ missing a pharmacy refill for anti-hypertensive, anti-diabetic, and/or anti-lipid agents	Proctor’s Outcomes for Implementation Research	• *# and % of participants with evidence of missing a pharmacy refill for a documented cardiometabolic NCD at 0, 12, and 24 months*
Feasibility	The extent to which a new treatment, or an innovation, can be successfully used or carried out within a given agency or setting	CFIR, Proctor’s Outcomes for Implementation Research	• *Average (mean) Feasibility of Intervention Measure (FIM) score after TASKPEN implementation at* ≥ *6 months from TASKPEN introduction* • *Perceptions at* ≥ *6 months from TASKPEN introduction among interview and FGD participants regarding the extent to which TASKPEN can be successfully carried out in their particular clinical setting*
Appropriateness	Perceived fit, relevance, or compatibility of the innovation or evidence-based practice for a given practice setting, provider, or consumer; and/or perceived fit of the innovation or evidence-based practice to address an issue	CFIR, Proctor’s Outcomes for Implementation Research	• *Average (mean) Intervention Appropriateness Measure (IAM) score after TASKPEN implementation at* ≥ *6 months from TASKPEN introduction* • *Perceptions at* ≥ *6 months from TASKPEN introduction among interview and FGD participants regarding the fit of TASKPEN to address co-morbid cardiometabolic NCDs among PLHIV accessing health services in routine HIV care settings*
Sustainability	Extent to which a recently implemented practice is maintained and/or institutionalized within a service setting’s ongoing, stable operations	Proctor’s Outcomes for Implementation Research	• *Average (mean) Clinical Sustainability Assessment Tool (CSAT) score after TASKPEN implementation at* ≥ *6 months from TASKPEN introduction* • *Perceived integration or institutionalization of the TASKPEN intervention as a routine practice, and perceived alignment with MOH policy as assessed through interviews and focus groups at* ≥ *6 months from TASKPEN introduction*
Cost-effectiveness	Financial impact of an implementation effort and the relative value in quality adjusted life years save of the intervention state over the standard of care state	Proctor’s Outcomes for Implementation Research	• *Incremental cost-effectiveness ratios (ICERs) at 0, 12 and 24 months*

### Randomization and masking

The trial biostatistician will use a computer-generated allocation algorithm in Stata (StataCorp, College Station, TX, USA) to randomize clusters to a time point of implementation and conceal timing from the facilities as well as the trial team until a few weeks before the implementation date. After implementation, the intervention will be unblinded to trial participants, care providers, and data collectors. We will use a similar computer-generated algorithm to randomly select 4 facilities (1 from each block) for the nested cohort. No summary reports on the primary outcome, dual HIV/NCD control, will be shared with any clinic or study staff in such a way that it could impact questions of equipoise.

### Sample size

The sample size calculation (Table 5) was based on comparisons of the primary clinical effectiveness outcome of dual control during the control and intervention periods. The control and intervention proportions are assumed to be 0.30 and 0.36, respectively, based on previous studies from Zambia [41, 42] suggesting high viral suppression (i.e., 95%) but poor (i.e., ~ 30%) control of cardiometabolic NCDs (0.32 * 0.95 = 0.30) [42, 43]. With these assumptions, and using the two-sided Wald Z-Test as the test statistic, an incomplete stepped-wedge cluster-randomized design with 5 time periods (T0-T4), 4 steps, 12 sites, and an average of 81 participants per cluster per time period achieves 80.417% power to detect a 6% absolute difference in the proportion of patients with dual control between control and intervention periods. This approach requires a calculated sample size of 4,860 participants. We will inflate the calculated sample size to allow for survey non-response and nested cohort recruitment. For non-response, we assume that ~ 5% of participants will have a missing lab parameter required for outcome ascertainment, and so we will enroll 85 individuals per cluster per time period, for a total of 5,100 participants. To recruit our nested cohort, we will need to enroll 200 participants at each of the 4 nested cohort sites during the baseline (or T1) surveys. With this rationale for sample size inflation, we anticipate a final total sample size of 5,560 survey participants.

**Table 5:** Statistical power at different sample size assumptions

	**Design**	**Clusters**	**Size**	**Size**	**Proportion**	**Proportion**	**Difference**		
**Power**	S/T/R	K	M/m	N	P1	P2	D1	ICC	Alpha
***0.80417***	*4/5/1*	*12*	*405/81*	*4860*	*0.36*	*0.3*	*0.06*	*0.2*	*0.05*
0.80198	4/5/1	12	295/59	3540	0.37	0.3	0.07	0.2	0.05
0.80181	4/5/1	12	225/45	3270	0.38	0.3	0.08	0.2	0.05

S = # of steps in the study design, S = T-1; T = # of time periods, including the baseline, T = S + 1; R = number of clusters switching from control to treatment at each step; K = Total number of clusters to be randomized, K = S*R; M = average number of participants per cluster, M = mx*T; m = average number of participants per cluster per time period, M/T; P1 = treatment proportion, assuming the alternative hypothesis; P2 = control proportion; D1 = Difference assuming the alternative hypothesis (H1); ICC = intracluster correlation coefficient

For the nested cohort, we will enroll a consecutive sample of baseline (and T1, if necessary) survey participants found to have a cardiometabolic NCD or risk factor through the survey. Assuming we enroll 2,500 survey participants at times T0 and T1 across all sites, that ~ 800 come from the 4 nested cohort sites, that approximately 38% of these (based on pilot data) meet nested cohort eligibility criteria, and that cohort loss to follow up will be about 5%, we will aim to enroll 80 nested cohort participants per site or 320 total nested cohort participants. A paired design will be used to test whether there is a difference in the proportion of nested cohort participants achieving dual control between the intervention and control periods. With 320 pairs, we will have > 80% power to detect a McNemar odds ratio of 1.47, or a difference between two paired proportions of 0.14 (i.e., 0.44 in those exposed to TASKPEN and 0.3 among the unexposed). The sample size calculations were computed using PASS 2022, version 22.0.3.

### Trial monitoring

Annual trial monitoring will be conducted by an external Research Coordinating Center to ensure that study procedures comply with good clinical practice and Zambian and international regulatory guidelines. All study staff will be responsible for the timely identification, reporting, assessment, and management of adverse events (AEs) according to the study protocol and standard operating procedures. Any unexpected AEs and serious adverse events will be reported in a timely fashion to the overseeing IRBs and an independent Data Safety Monitoring Board (DSMB). The DSMB is comprised of eight members with diverse research expertise and is responsible for trial oversight, monitoring study data, and protecting participant safety. The DSMB will review interim safety analyses and has authority to terminate the trial early, if needed. Any modifications to the protocol will be communicated to the DSMB, as well as the sponsor, IRBs, and trial registry.

### Study procedures for completion of aim 1

#### Recruitment and informed consent

For the patient surveys, we will recruit a simple random sample of PLHIV scheduled to access routine HIV services at the study sites during the times planned for each survey. We will work with clinic staff to review routinely available appointment records kept at each site, including routine clinic locator information containing patient phone numbers, to create a list of every patient with an upcoming appointment. We will then select every N^th^ patient with an upcoming appointment, who is not known to be dead or to have transferred out of the clinic (but including those potentially lost to follow up or experiencing treatment interruption), and contact them by phone using a standardized study recruitment script. If we do not meet our pre-specified daily site-level recruitment targets using the aforementioned approach, we will supplement with in-person recruitment. In person recruitment will proceed by asking every N^th^ patient present at the clinic during the surveys if they would be interested in learning more about the study during patient waiting times. After recruitment, interested persons will be offered a chance to participate after completing informed consent procedures (Additional file 2) with a research assistant or other trained study staff member.

For the nested cohort, we will contact a consecutive sample of all survey participants who we identify with an eligible cardiometabolic NCD or risk factor through the baseline survey (or T1 survey if recruitment is not completed at T0) at four randomly selected sites and invite them to be followed longitudinally following written informed consent.

To achieve greater balance of biological sex, if more than 60% of enrolled survey or nested cohort participants are of one sex, then we will attempt to preferentially sample from the other sex until that percentage falls to or below 60%.

#### Study instruments and data collection

Data collection tools and instruments described in this protocol have been piloted and refined through the preceding formative protocol (NCT05005130), and include a patient survey, lab result case reporting form (CRF), WHO QOL HIV Bref questionnaire [44], and patient economic expenses questionnaire. For our patient surveys, data will be collected at specified time points over a period of about 30 months (i.e., T0–T4). Study staff will administer a one-time patient survey to participants using an electronic tablet to collect information on: socio-demographic factors; medical history; alcohol and tobacco use; ART and NCD medication regimens and adherence; dietary and exercise habits; purchase of medications in the private sector; and healthcare utilization. The patient survey is provided as an additional file (see Additional file 1). As part of the survey, participants will also have anthropometric and cardiovascular measures taken, have a random/fasting blood glucose checked, and undergo phlebotomy for cholesterol and hemoglobin A1c testing (if the random/fasting blood glucose is elevated). We will assess viral load through review of viral load test results available in the routine medical record. In Zambia, HIV viral load is offered through the national program at 6 and 12 months after ART start and then yearly thereafter. During each survey, if we note that survey participants did not have a routine viral load test result within the preceding ~ 3–6 months, we will provide “catch up” HIV-1 viral load testing through the study. Following completion of the survey, participants who are not eligible for the nested cohort will be exited.

Detailed data collection for the nested cohort will continue longitudinally every 6 months from enrollment until trial end. To ensure nested cohort retention, study peer educators will trace by phone or in person any participants who miss a study visit or deviated from the nested cohort procedures summarized in Table 6.

**Table 6:** Nested Cohort Visit Schedule and Procedures

Nested Cohort Procedures	Visit Month				
	**0**	**6**	**12**	**18**	**24**
Cohort enrollment visit	•				
Cohort follow-up visit		•	•	•	•
Patient survey administration	•	•	•	•	•
Medical record review and abstraction	•	•	•	•	•
Anthropometric and cardiovascular measures taken	•	•	•	•	•
WHOQOL-HIV-Bref-Zambia quality of life questionnaire^a^	•		•		•
Patient economic questionnaire (part of costing study)^a^	•		•		•
Patient implementation questionnaire (including AIM, FIM, IAM)^a^	•		•		•
Random/ fasting blood glucose (RBG/ FBG)	•	•	•	•	•
Screening Hemoglobin A1c (if RBG/ FBG elevated)	•	•	•	•	•
Monitoring Haemogloblin A1c (for diabetics and pre-diabetics)	•	•	•	•	•
Lipid Profile^a^	•		•		•
HIV-1 VL (if not documented in medical record within past ~ 3–6 months)^a^	•	•	•	•	•

*AIM* Acceptability of intervention measure, *FIM*  Feasibility of intervention measure, *IAM*  Intervention appropriateness measure, *RBG*  Random blood glucose, *FBG* Fasting blood glucose, *VL* Viral load

^a^Procedure may be done at the 18-month visit in lieu of the 24-month visit for nested cohort participants enrolled after the T1 survey

#### Data management

Study-specific survey and cohort data will be merged with electronic routine data to create a comprehensive, and secure database in REDCap (Nashville, Tennessee, USA). Data in the trial database will be organized by participant identification number assigned to each participant that will then serve as the lone study identifier to protect participant confidentiality. The study database will be encrypted and password protected and backed up daily on a secure, encrypted server restricted to authorised study staff only. The study data manager will conduct regular database QA/QC queries for extreme, missing, and illogical values.

#### Learn as you GO (LAGO) methodology

To allow for adaptation and refinement of the TASKPEN package as the trial progresses, we will use the novel ‘Learn As you GO’ or ‘LAGO’ methodology [45]. Using LAGO, at the trial mid-point (i.e., after the T2 midline survey around 12 months into trial implementation), the data collected up to that point will be used to optimize the presence and dose of pre-specified components of the TASKPEN package. Then, at the start of introduction of TASKPEN to clusters 7 and 8, a modification to the TASKPEN intervention will be suggested to increase TASKPEN uptake.

#### Statistical methods for aim 1

The primary analysis will be performed on the intention-to-treat (ITT) population, applying sampling weights based on our sampling approach. Continuous and categorial variables will be summarized using descriptive statistics. Mixed-effect logistic regression modeling will be used to estimate the effect of the intervention on dual control of HIV and cardiometabolic NCDs, adjusting for potential confounders and covariates imbalanced between the intervention and SOC populations. We plan to adjust for age, gender, time on ART, ART regimen, status in the HIV program, having new (i.e., identified through the study) versus an established (i.e., previously known) cardiometabolic NCD, and duration or “dose” of exposure to TASKPEN, among other variables identified by our directed acyclic graph. In planned sensitivity analyses, we will exclude participants who were not meaningfully exposed to the control or intervention and examine effects according to various durations of intervention exposure. We will also examine results after including uncontrolled dyslipidemia in our composite definition of dual HIV-NCD disease control.

Continuous outcomes for systolic blood pressure, diastolic blood pressure, 10-year ASCVD risk score, hemoglobin A1c, low-density lipoprotein (LDL) and total cholesterol, and quality of life scores will be analyzed in an analogous way using mixed-effects linear regression models. We will adjust for changes in age observed over the study period that might affect ASCVD risk scores in the model. The proportion with disease control in the intervention versus control periods for individual conditions like hypertension and diabetes will be compared using mixed-effects logistic regression model, adjusted for potential confounders. All analyses will be performed using Stata 18 MP8 (StataCorp, College Station, TX, USA) or R 4.2.2.

For the nested cohort, we will use random-effects binomial regression modelling to assess the relationship between the TASKPEN package and binary and continuous outcomes of interest. The intervention status of a cohort participant will correspond to the intervention status of the cluster of the participant. Using a panel data analytic technique, we will adjust for the temporality in outcome measurement. For continuous outcomes, we will use a random-effects linear regression model.

Multiple imputation will be used to handle missing data. In cases where we want to account for one variable at a time, we will apply sampling weights based on recruitment of survey participants into the nested cohort. Measurements from the nested cohort will be used to augment measurements in the survey by upweighting participants in the cohort to represent all eligible participants who were missing a value in the survey. We will use a similar approach to make inferences about the distributions of cardiometabolic outcomes for the total clinic population at our study sites.

To compare patient-level HIV outcomes, we will report differences and ratios with accompanying 95% confidence intervals for retention, adherence, and viral suppression according to definitions in Table 3.

### Study procedures for completion of aim 2

To assess implementation outcomes and explore explanatory mechanisms for why the TASKPEN package succeeds or fails in the trial, we will collect the following data embedded in the larger pragmatic trial: questionnaires with patients and providers, including NPHWs; in-depth interviews (IDIs) with patients; focus group discussions (FGDs) with NPHWs and CHWs supporting TASKPEN delivery; and key informant interviews (KII) with stakeholders.

#### Study population

For aim 2, the study population will encompass patients exposed to the TASKPEN package as trial survey or cohort participants, as well as stakeholders and implementation actors involved in TASKPEN delivery.

#### Data collection

Data collection activities for aim 2 will involve a combination of non-human subjects research (NHSR) and implementation science research activities (Table 7). Two NHSR activities will take place, the first being structured observations and the second being a costing study for the cost-effectiveness analysis.

**Table 7:** Summary of data collection activities using mixed methods for Specific Objective #2

Method	Activity Type	Participant inclusion criteria	Total Participants	Approximate time for each activity
Structured Observations	*Non-human subjects research*		0 (Non-participatory)	1–2 h
Costing survey	*Non-human subjects research*	HIV-positive adults ≥ 18 years of age who had received HIV and/or NCD services at a TASKPEN study site, OR ≥ 18 years of age and a facility-level healthcare provider or manager at facility, district, provincial, or national level in Zambia, and generally familiar with HIV and/or NCD-related issues	0 (Non-participatory)	1.5 h
Focus group discussions	*Research*	≥ 18 years of age; a NPHW or community health worker (CHW)/ lay health provider involved with TASKPEN or integrated HIV/NCD service delivery; and generally familiar with HIV and/or NCD service delivery at their facility	72 – 96 NPHW (1 FDG at each site and 6–8 per FGD)	1.5 h
	*Research*		72 – 96 CHW/lay health providers (1 FDG at each site and 6–8 per FGD)	1.5 h
	*Research*		144 – 192 (all FDG participants)	15 min
In-depth interviews	*Research*	HIV-positive adults ≥ 18 years of age who were survey and/or cohort participants at a TASKPEN study site	~ 40 (3–4 per site)	~ 1 h
Key informant interviews	*Research*	≥ 18 years of age; a facility-level ART, differentiated service delivery (DSD), outpatient department (OPD), or relevant clinical leader/ manager/ in-charge, or policy maker at district, provincial, or national level in Zambia; and generally familiar with HIV and/or NCD-related issues in their community	~ 12 (1 per site)	~ 1 h
*Total Mixed Methods Participants*			** ~ 196 – 244**	

#### Mixed methods study instruments

A summary of all data collection tools for completion of aim 2 are presented in Table 8.

**Table 8:** Summary of data collection tools employing mixed methods for completion of aim #2

Data collection tool title	Structured Observation	IDI	FGD	KII	Costing
Structured Observation Guide	•				
Post FGD/ KII Implementation Questionnaire			•	•	
Non-Physician Health Worker Focus Group Discussion (FGD) Guide			•		
Community Health Worker Focus Group Discussion (FGD) Guide			•		
Participant In-depth Interview Guide		•			
Key Informant Interview Guide TASKPEN				•	
Facility Costing Survey					•
Patient Clinic Flow Time & Motion Tool					•
Health Worker Time & Motion Direct Observation Tool					•
Health Worker Time & Motion Self-Report Tool					•

Structured observations acknowledge the importance that health system actors play in delivering integrated HIV/NCD care. During observations, trained observers will observe patient flow and other elements of day-to-day clinic operations. Structured observations will be conducted twice at each site—once before and once after introduction of TASKPEN. Data collected on the guides will be formalized into research memos and will contribute to building a picture of integrated HIV/NCD service delivery informed by CFIR model domains and constructs [33].

Qualitative guides for FDG, IDI, and KII were developed and refined during the study pilot. All guides were informed by the CFIR and feedback from stakeholders using human-centred design approaches, and have been developed in English and translated into *Bemba* and *Nyanja*, the two most commonly spoken languages in Lusaka. An implementation questionnaire will be administered to participants at the end of each KII and FGD. The implementation questionnaire features the FIM, AIM, and IAM, [46] as well as the Program Sustainability Assessment Tool (https://www.sustaintool.org/csat/), among other tools [47].

#### Costing data collection

Costing data collection will have three components: 1) a patient economic questionnaire in the nested cohort; 2) an administrative costing data review; 3) a facility costing survey. For the administrative costing component, we will review clinic administrative records at the 12 study sites to evaluate patient clinical service use and associated costs.

For the facility costing survey component, we will conduct a costing survey at all 12 study sites before and after TASKPEN introduction involving ~ 30 Ministry of Health (MOH) informants and observations of patients and providers. The facility costing survey will observe:

- The NCD service components accessible at ART and DSD clinics;
- The proportion of human resources trained to deliver TASKPEN;
- Distribution of overheads across various centers, and attribution to the TASKPEN package;
- Salaries of all staff cadres involved in the TASKPEN package/ integrated HIV/NCD services;
- Time spent by patients waiting, preparing for, or receiving integrated HIV/NCD services (patient time and motion)
- Time spent by providers waiting, preparing for, or delivering TASKPEN (provider time and motion)
- The quantity of material inputs and costs utilized in implementation of the TASKPEN intervention;

The patient time and motion survey will involve observing ~ 20–25 patients per facility at each time point before and after the intervention (i.e., ~ 360–400 total) for waiting times, frequency of visits, and use of facility level services as they move throughout the facility. For providers, the time and motion survey will involve observing and documenting the start and end times of service delivery-related activities for ~ 5–7 providers per facility at each time point (i.e., ~ 120–160 total). We will observe various staff cadres at each facility. All these data sources will be combined in the final facility costing survey for each facility.

#### Outcomes

We will evaluate implementation outcomes of reach, as well as adoption and implementation patterned after the RE-AIM framework [34, 35] (Table 4). Quantitative metrics adapted from RE-AIM will be enriched with qualitative assessments according to CFIR and Proctor’s Conceptual Model of Implementation Research [48].

#### Statistical methods overview

We will use simple descriptive statistics to describe reach, adoption, implementation, and fidelity according to the definitions provided in Table 4, and calculate means and measures of dispersion for quantitative measures of acceptability, feasibility, appropriateness, and sustainability.

#### Costing analysis

The costing analysis will include costs for the following items: (i) staff time, (ii) personnel costs, (iii) medical and nonmedical consumables, (iv) type and number of laboratory tests, (v) capital allowances and overheads, and (vi) administrative costs. We will present aggregated total costs and unit costs for integrated and SOC service delivery. Estimation of patient economic costs will be measured by: (i) direct patient out-of-pocket expenses from the patient economic questionnaire; and (ii) indirect costs defined as value of lost production time.

For the cost-effectiveness analysis, we will develop a simplified Markov state-transition model that represents care pathways for the TASKPEN package and SOC. Cost-effectiveness for the TASKPEN package relative to the SOC will be evaluated based on willingness-to-pay thresholds. The primary cost-effectiveness outcome will be assessed based on cost per patient with dual control. Secondarily, we will model the conversion of cardiometabolic disease control and viral suppression into disability adjusted life years (DALYs) averted, reflecting Zambia’s age and disease adjusted life-expectancy, and disability weights [49]. Health systems costs will also be tracked for participants from the point of study enrollment, and will be tallied based on expected health services utilization for each care pathway.

#### Qualitative data analysis

**Fig. 3:**
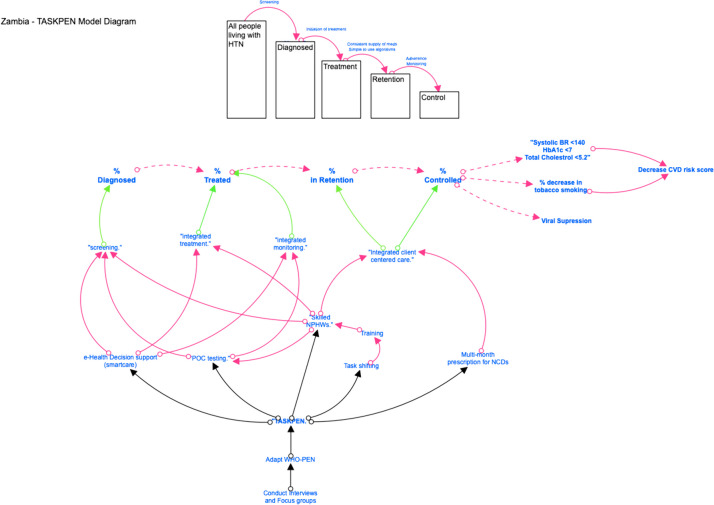
Conceptual model of change for TASKPEN intervention, showing how intervention components ultimately can lead to dual disease control and reduced CVD risk

The qualitative data analysis is intended to elucidate the mechanisms, barriers and facilitators, and contextual factors that influenced our clinical and implementation outcomes. Content analysis guided by CFIR, RE-AIM, and our conceptual model (Fig. 3) will be used to code, analyze, and interpret our data [46]. Selected domains and constructs of the CFIR have been featured in our qualitative guides [33, 46], including: complexity, patient needs and resources, perceived healthcare worker challenges, structural characteristics, knowledge and beliefs, planning, and executing. Once all codes have been finalized and themes developed, we will compare them across different participant types and qualitative data collection methods using matrices [46]. We will apply principles of triangulation, negative case analysis, and respondent validation to minimize bias in the qualitative analysis.

#### Results dissemination

TASKPEN results will be disseminated locally to participants and partner communities and organizations in Zambia. Results will also be shared with the scientific community through conferences, journal publications, and reporting in ClinicalTrials.gov.

## Discussion

While mounting data describe an aging global cohort of PLHIV with a growing burden of cardiometabolic NCDs and the evidence-based tools available to combat these NCDs, relatively little is known about how to deploy these tools in real-world clinical practice settings in SSA. Moreover, few studies have described the diverse local contexts, available HIV service delivery platforms, and models of service integration that can support the global HIV response as it moves toward universal primary care for PLHIV. The TASKPEN study is designed to address these urgent knowledge gaps by contributing new evidence on how best to integrate NCD and HIV services in African settings with high HIV/NCD burden and limited resources, and on how NPHWs can contribute to service delivery in these settings. TASKPEN also advances a new concept of ‘dual control’ for HIV and NCDs, which may help HIV programs think beyond viral suppression to more holistic and person-centred metrics for the health and wellbeing of PLHIV.

The TASKPEN package is designed to be feasible for urban, PEPFAR-supported HIV care settings in Zambia. The EBP components were selected to be practical and intentionally leverage existing PEPFAR and MOH health system investments, including for the national EMR, health worker capacity building, pharmacy and laboratory systems, and patient-centred care innovations like multi-month medication dispensing. The greatest challenge for TASKPEN feasibility may be the need to restructure clinical workflows to create one-stop shops where HIV and NCD services for PLHIV can be provided together using the same space, providers, and health record, which is addressed directly by our multifaceted implementation strategy. Our strategy, with service site reorganization and integration at its core, and peripheral components of task-shifting, practice facilitation, and upgrading equipment, physical structures, and medical information systems is being evaluated in hopes that it can overcome the known barriers that have stymied integrated NCD care in the past.

Emerging evidence from high-income and lower-middle income settings support integrating prevention, management, and surveillance of NCDs into other models of chronic care [20, 50]. Integrated care requires a multidisciplinary approach that engages various health workers, and critical to this approach in resource-constrained settings is task-shifting. The task-shifting strategy is premised on the fact that most health facilities in LMICs have inadequate numbers of trained health workers—and especially doctors—to meet the needs of the population. Therefore, engaging CHWs, nurses, and other NPHWs to lead cardiometabolic NCD care is a cornerstone of our multifaceted implementation strategy and has been shown to improve blood pressure control in general, non-HIV populations in India, Ghana, and Nigeria [51–54].

Although the TASKPEN package has underpinnings in prior clinical research and formative work from Zambia, demonstrating its impact will require robust clinical and implementation outcome measurement. To accomplish this, we have selected a pragmatic trial design and powered it around a primary outcome of ‘dual control’ focused on properly treating *both* HIV and cardiometabolic NCDs. We have deliberately done this to capture the hypothesized effects of the TASKPEN package on multiple aspects of health of PLHIV, and to advance the public health narrative about what should matter and be measured by HIV treatment programs. Thanks to now widely available and efficacious ART regimens, viral suppression alone, while an important biomarker, need not be the ultimate criterion for clinical or programmatic success. Rather, a measure like dual control that more closely approximates more distal and meaningful clinical outcomes like mortality, and attempts to estimate effects on common comorbidities may better focus researchers’, clinicians’, and implementers’ attention on the interventions most likely to prolong life and address multi-morbidity for PLHIV. Indeed, in an era where the third 95 is in sight, preventing premature death from any cause, including cardiometabolic NCDs, and supporting PLHIV to access chronic care that improves quantity *and* quality of life should be the ultimate goal.

To rigorously measure dual control and other study outcomes, we have incorporated several analytical innovations to overcome methodological limitations inherent to stepped-wedge trials. First, we have added a prospective cohort at four randomly selected study sites to overcome limitations of serial cross-sectional outcome ascertainment for outcomes like cardiovascular disease risk. Second, we intend to estimate the effects of the TASKPEN package on dual control among the total clinic population—not just among study participants—to improve study generalizability and better understand how TASKPEN may influence performance in the national HIV program. Therefore, we will weight data from the patient surveys and nested cohort, which are both sub-populations sampled from each site, to enrich routine medical data available at the sites and overcome any missingness in routinely collected data. Third, we will employ the LAGO method at the study midline to refine the dose and intensity of selected components of our multi-faceted implementation strategy. Finally, we will conduct cost-effectiveness analyses, as well as mixed methods evaluations applying established implementation science theory, to understand patient- and health system-level mechanisms and mediators of HIV/NCD integration during the trial.

### Implications

Findings from this study can inform discrete, actionable, and context-specific recommendations to integrate cardiometabolic NCD care into Zambia’s national HIV treatment program. While the TASKPEN study focuses on cardiometabolic NCDs, the multifaceted implementation strategy studied will be relevant to other NCDs in PLHIV. Finally, it is expected that the trial will generate new insights that enable delivery of more person-centred and integrated HIV/NCD care, which may promote improved long-term outcomes for PLHIV in Zambia and elsewhere in the region.

### Supplementary Information

Additional file 1. Includes the main data collection survey tool for the trial.

Additional file 2. includes the informed consent form for the main trial survey.

## Data Availability

De-identified datasets along with the accompanying statistical code will be made available upon request to the National Health Research Authority and study multiple principal investigators after the publication of the main findings. We will require interested parties to submit a data request in writing. Once approved, data transfer agreements will be developed. All data transfers comply with HIPAA regulations.

## Abbreviations

ART: Antiretroviral therapy
CHW: Community health worker
CVD: Cardiovascular disease
EBP: Evidence-based practice
LMIC: Low- and middle-income country
NCD: Non-communicable disease
NPHW: Non-physician health workers
PLHIV: People living with HIV
SSA: Sub-Saharan Africa
WHO: World Health Organization
WHO PEN: WHO *Package of Essential **Noncommunicable** Disease Interventions for Primary Care*
