# Supplementary material for: Evaluating a multifaceted implementation strategy and package of evidence-based interventions based on WHO PEN for people living with HIV and cardiometabolic conditions in Lusaka, Zambia: protocol for the TASKPEN hybrid effectiveness-implementation stepped wedge cluster randomized trial
Source: Implement Sci Commun. 2024 Jun 6;5:61. doi: 10.1186/s43058-024-00601-z (PMC11155136; doi:10.1186/s43058-024-00601-z)
Supplement: Supplementary file 1 — Additional file 1. Includes the main data collection survey tool for the trial. [file 43058_2024_601_MOESM1_ESM.pdf]

TASKPEN UH3 Patient Survey v1.1

|___|___|___|-|___|___|___|___| |___|___| / |___|___|/ |___|___|___|___|

Site Code - Participant ID DD/MM/YYYY

Patient Survey

Instructions:

- *Please complete this CRF for all participants who have enrolled in the study, by speaking with the participant directly and/or checking the patient's SmartCare/ART/NCD patient file and/or relevant register(s) at the facility.*
- By "**study labs**" we mean routine labs of interest drawn by clinic staff or study-specific labs drawn by study staff.
- Results for lab tests drawn or initiated by the study may be found from study registers and forms, SmartCare files, the Laboratory Information Management System (LIMS), and DiSA.
- This form should be completed no later than 7 days after labs are drawn for cardio-metabolic tests and no later than 21 days after a viral load sample is taken.
- Please confirm with all relevant study and clinic staff, as well as by reviewing the participant's file in SmartCare and LIMS that no results are actively pending prior to indicating that "NO" test was done.
- If any test result is pending or cannot be found in the participant record, flag the record, and refer to the Study/ QA/QC nurse for follow-up and possible repeat testing.
- The date format is DD/MM/YYYY e.g., 01/06/2021 for the 1st of June in the year 2021.
- If, after exhaustive follow up, a date is not documented, enter "99/99/9999" in the date field.
- If viral load is unknown, enter 999,999,999
- If an entry field has more digits than an answer requires, please use leading "0"s to ensure the field is filled completely.
- If there are questions about administration of the survey, please contact the Study Coordinator.

TO BE COMPLETED BY: RESEARCH ASSISTANTS, STUDY FELLOWS, OR STUDY NURSES

PART A: SOCIAL DEMOGRAPHICS

1. Date of birth |___|___| / |___|___|/ |___|___||___|___|
   DD/MM/YYYY

2. Gender of patient |___|___|
   (00) Male
   (01) Female
   (02) Other

3. Highest level of Education Completed |___|___|
   (00) No formal schooling
   (01) Primary School
   (02) Secondary School Certificate
   (03) Undergraduate Degree
   (04) Post-graduate Degree
   (98) Prefers not to answer
   (97) Participant does not know

4. Current Marital Status of participant |___|___|
   (00) Never Married
   (01) Currently Married
   (02) Separated
   (03) Divorced
   (04) Widowed
   (05) Common Law/ Cohabitating
   (98) Prefers not to answer
   (97) Participant does not know

|___|___|___|-|___|___|___|___| |___|___| / |___|___|/ |___|___|___|___|
Site Code - Participant ID DD/MM/YYYY

PART B: PHYSICAL EXAMINATION / CARDIOVASCULAR AND ANTHROPOMETRIC MEASURES

5. Systolic Blood Pressure (SBP) **Measurements taken after 2-minute intervals**

SBP 1 |___|___|___|mmHg

SBP 2 |___|___|___|mmHg
Taken 2 minutes after the first measurement

SBP 3 |___|___|___|mmHg
Taken 2 minutes after the second measurement

6. Diastolic Blood Pressure (DBP) **Measurements taken after 2-minute intervals**

DBP 1 |___|___|___|mmHg

DBP 2 |___|___|___|mmHg
Taken 2 minutes after the first measurement

DBP 3 |___|___|___|mmHg
Taken 2 minutes after the second measurement

7. Pulse Rate **Measurements taken after 5-minute intervals** (in beats per minute, bpm)

Pulse Rate 1 |___|___|___|bpm

Pulse Rate 2 |___|___|___|bpm
Taken 5-minutes after the first measurement

Pulse Rate 3 |___|___|___|bpm
Taken 5-minutes after the second measurement

8. Height |___|___|___|. |___|cm

9. Weight |___|___|___|. |___|kg

10. Hip Circumference |___|___|___|. |___|cm

11. Waist Circumference |___|___|___|. |___|cm

12. Date of most recent blood sample collection for viral load:
|___|___| / |___| ___|/ |___|___|___|___|
DD/MM/YYYY

12a. Date of most recent viral load results received: |___|___| / |___| ___|/ |___|___|___|___|
DD/MM/YYYY

12b. Most recent viral load result: |___|___|___|, |___||___|___|, |___|___|___| Copies/ml

Note: If result is "undetectable," enter "01"; If NO viral load has been recorded, write "999,999,999"

13. Date of most recent blood draw for blood sugar test:
|___|___| / |___| ___|/ |___|___|___|___|
DD/MM/YYYY

13a. Please indicate if the blood draw is for a ***fasting*** or ***random*** blood glucose.
(00) **Random** |___|___| (01) **Fasting** |___|___|

Before filling out this question, remember to ask the participant if they had taken any food and/or sugary drinks before coming to the facility.

13b. Date of most recent blood sugar test results received: |___|___| / |___| ___|/ |___|___|___|___| *DD/MM/YYYY*

13c. Most recent blood sugar test result: |___||___|. |___| *g/dL*

14. Date of most recent blood draw for HbA1c test: |___|___| / |___| ___|/ |___|___|___|___| *DD/MM/YYYY*

14a. Date most recent HbA1c test results received: |___|___| / |___| ___|/ |___|___|___|___| *DD/MM/YYYY*

14b. Most recent HbA1c test result: |___|___|. |___| %

15. Date of most recent blood draw for low density lipoprotein/ LDL: |___|___| / |___| ___|/ |___|___|___|___| *DD/MM/YYYY*

15a. Date most recent LDL test results received: |___|___| / |___| ___|/ |___|___|___|___| *DD/MM/YYYY*

15b. Please indicate if it was a ***fasting*** LDL (00) No |___|___| (01)Yes |___|___|

15c. Most recent LDL test result: |___||___|. |___| mmol/*L*

PART C: BASIC INFORMATION

The following questions should be asked directly to the patient <u>with</u> their Smart Care File and/or NCD record on hand for reference. If the patient is unsure about any question, mark the unanswered question for review and then consult their Smart Care/ medical file once the patient has left. If information is still unavailable, after having consulted with clinic staff, mark the answer as unsure or not documented as appropriate.

16. Does the patient currently smoke tobacco (any tobacco smoking in the last 30 days)? |___|___|
(01) Current Smoker
(02) Former smoker
(03) Never smoker
(98) Prefers not to answer
(97) Participant does not know
(88) Other specify;________________________

If the participant is a current smoker or former smoker, go to 16a. If no, go to 17.

17a. Number of cigarettes smoked per day, on average, in the last 30 days. |___|___|___|

17. Does the patient currently drink alcohol (any alcohol consumption in the last 30 days)? |___|___|
(00) No
(01) Yes
(98) Prefers not to answer
(97) Participant does not know

17a. How often do you have a drink containing alcohol? |___|___|
(00) Never
(01) Monthly or less
(02)2-4 times a month
(03)2-3 times a week
(04)4 or more times a week
(98) Prefers not to answer
(97) Participant does not know

17b.How many standard drinks containing alcohol does the patient have on a typical day? |___|___|
A standard drink Includes one bottle of beer, one glass of wine, one cup of homebrewed drink, or one shot of spirits
(00)1 or 2
(01)3 or 4
(02)5 or 6
(03)7 to 9
(04)10 or more
(98) Prefers not to answer
(97) Participant does not know

17c. How often does the patient have six or more drinks on one occasion? |___|___|
(00) Never
(01) Less than monthly
(02) Monthly
(03) Weekly
(04) Daily or almost daily
(98) Prefers not to answer
(97) Participant does not know

PART D: HIV CHARACTERISTICS

Instructions: If self-reported, all data should be checked against the SmartCare file.
****NOTE: Confirm date in Smart Care File, if possible***
****For patient responses:***
****If the exact day is unknown, place "99" above "DD"***
****If the exact month is unknown, place "99" above "MM"***
****If the exact year is unknown, ask the patient to provide the best estimate for how long ago they were diagnosed with HIV and calculate accordingly.***

18. When were you first diagnosed with HIV? |___|___| / |___|___|/ |___|___|___|___|
DD/MM/YYYY

19. When were you initiated on ART? |___|___| / |___|___|/ |___|___|___|___|
DD/MM/YYYY

If the date for blood sample collection is not available, use date result was reported or received at the facility

20. Has the participant taken at least one ARV pill at any time in the last 30 days? |___|___|

(00) No
(01) Yes
(98) Prefers not to answer
(97) Participant does not know

21. When was the date of the last documented ARV <u>prescription</u> collection/ pharmacy visit?
|___|___| / |___|___|/ |___|___|___|___|
DD/MM/YYYY

If day, month or year are not documented write"99"/"99"/"9999" as required

22. Which ART regimen was the participant prescribed on their most recent visit prior to study enrolment? |___|___|

(00) Atripla (TDF + FTC/3TC + EFV)
(01) TLD (TDF + FTC/3TC + Dolutegravir/DTG)
(02) TafED (TAF + FTC/3TC + Dolutegravir/ DTG)
(03) TDF + FTC/3TC + ATV-r
(04) AZT + FTC/3TC + DRV-r
(05) TDF + FTC/3TC + LPV-r
(06) ABC + FTC/3TC + LPV-r
(07) AZT + FTC/3TC + LPV-r
(08) AZT + FTC/3TC + ATV-r
(09) ABC + FTC/3TC + Dolutegravir/ DTG
(88) Other regimen, specify: ___________________
(99) Not Documented
(98) Prefers not to answer
(97) Participant does not know

23. How many days' ART pills were supplied to the participant on the last pharmacy visit above? |___|___|___|

24. In the past 7 days, has the participant physically had a supply/ refill of ARVs available to take? |___|___|
(00) No
(01) Yes
(98) Prefers not to answer
(97) Participant does not know

25. In the past 7 days, how many doses of ARVs has the participant missed? |___|___|
(00) None
(01) One
(02) Two
(03) Three
(04) Four or more
(98) Prefers not to answer
(97) Participant does not know

25a. If the participant missed at least one dose, what was the reason? |___|___|

(00) Chose not to take the medication due to side effects.
(01) Chose not to take the medication because couldn't take in private.
(02) Missed dose because participant had run out of medication
(03) Missed dose because participant forgot to carry the medication.
(98) Prefers not to answer
(97) Participant does not know
(88) Other reason, please specify: _______________________

26. In the last six months, did you miss the collection of your ARVs at this or another facility?
(00) No
(01) Yes
(98) Prefers not to answer
(97) Participant does not know
If No, skip to question 27.

26a. If yes, did you buy the ARVs from a private pharmacy during this time?
(00) No
(01) Yes
(98) Prefers not to answer
(97) Participant does not know
If No, skip to question 27.

26b. If yes, please tell us when and where you bought medications from a private pharmacy?

**Date of Visit**	**Name of Private Pharmacy**		**Number of days collected**
1. |___|___| / |___|___|/ |___|___|___|___| DD/MM/YYYY	(00) Link Pharmacy<br>(01) Cairo Pharmacy<br>(02) Medlink Pharmacy<br>(03) Jubilee Chemist<br>(04) Mega Pharmacy<br>(05) Local Pharmacy, name unknown<br>(88) Other, specify: _______	|___|___|	|___|___|___|
2. |___|___| / |___|___|/ |___|___|___|___| DD/MM/YYYY		|___|___|	|___|___|___|
3. |___|___| / |___|___|/ |___|___|___|___| DD/MM/YYYY		|___|___|	|___|___|___|
4. |___|___| / |___|___|/ |___|___|___|___| DD/MM/YYYY		|___|___|	|___|___|___|

27. Within the past 6 months, did you stop taking ARVs for more than 7 days for any reason (i.e., "stopped" means that they did not resume taking ARVs)?
|___|___|
(00) No
(01) Yes
(98) Prefers not to answer
(97) Participant does not know

27a. If "Yes", how long did you stay without taking your ARVs? |___|___|

(00) 0 – 7 days
(01) More than 7 days but less than 30 days
(02) One month (31-59 days)
(03) Two months (60-89 days)
(04) Three months (90-119 days)
(05) Four or more months (≥120 days)
(88) Other, specify: ___________________
(98) Prefers not to answer
(97) Participant does not know

27b. If you stopped ARVs for more than 7 days, what was the reason? |___|___|

(00) Side effects
(01) Advised to stop by clinician
(02) Medication was not available at the facility
(03) Could not afford to buy medication
(04) Started alternative medicine/myths in place of ARVs (i.e., water therapy)
(05) Taking traditional herbs/medication
(06) I was tired of taking ARVs.
(07) I lost my supply of ARVs.
(88) Other reason, specify: ____________
(98) Prefers not to answer
(97) Participant does not know

28. Do you know when you were last tested for Viral Load? |___|___|

(00) No
(01) Yes
(98) Prefers not to answer
(97) Participant does not know

29. Do you know whether your most recent Viral Load was undetectable or suppressed? |___|___|

(00) No
(01) Yes, suppressed.
(02) Yes, undetectable
(98) Prefers not to answer
(97) Participant does not know

30. When was the last documented Clinical Follow-up or Short Visit date at this ART centre before study enrolment?

|___|___| / |___|___|/ |___|___||___|___|
DD/MM/YYYY

If day, month, or year are not documented write"99"/"99"/"9999" as required

PART E: HIV MEDICATION ADHERENCE

31. Do you ever forget to take your HIV medication? |___|___|

(00) No
(01) Yes

(98) Prefers not to answer
(97) Participant does not know

32. Are you negligent/ forgetful at times about taking your HIV medication? |___|___|
(00) No
(01) Yes
(98) Prefers not to answer
(97) Participant does not know

33. When you feel better, do you sometimes stop taking your HIV medication? |___|___|
(00) No
(01) Yes
(98) Prefers not to answer
(97) Participant does not know

34. Sometimes if you feel worse when you take the HIV medication, do you stop taking it? |___|___|
(00) No
(01) Yes
(98) Prefers not to answer
(97) Participant does not know

35. I take my HIV medication only when I feel sick. |___|___|
(00) No
(01) Yes
(98) Prefers not to answer
(97) Participant does not know

36. It is unnatural for my mind and body to be controlled by HIV medication |___|___|
(00) No
(01) Yes
(98) Prefers not to answer
(97) Participant does not know

37. My thoughts are clearer on HIV medication. |___|___|
(00) No
(01) Yes
(98) Prefers not to answer
(97) Participant does not know

38. By staying on HIV medication, I can prevent getting sick. |___|___|

(00) No
(01) Yes
(98) Prefers not to answer
(97) Participant does not know

39. HIV medication makes me feel tired and sluggish. |___|___|

(00) No
(01) Yes
(98) Prefers not to answer
(97) Participant does not know

PART F: DISEASE DIAGNOSIS

40. Has the patient ***ever*** been diagnosed with or given medications for the following? Indicate "Yes," "No," or "Unsure / Not documented" for each item as follows:

a. Diabetes ("High Sugar") |___|___|

(00) No
(01) Yes
(99) Not documented
(98) Prefers not to answer
(97) Participant does not know

b. Psychiatric illness (like depression) |___|___|

(00) No
(01) Yes
(99) Not documented
(98) Prefers not to answer
(97) Participant does not know

c. Lung disease (Including history of TB) |___|___|

(00) No
(01) Yes
(99) Not documented
(98) Prefers not to answer
(97) Participant does not know

d. Kidney disease |___|___|

(00) No
(01) Yes
(99) Not documented

(98) Prefers not to answer
(97) Participant does not know

e. Liver disease |___|___|
(00) No
(01) Yes
(99) Not documented
(98) Prefers not to answer
(97) Participant does not know

f. Hypertension (High "BP") |___|___|
(00) No
(01) Yes
(99) Not documented
(98) Prefers not to answer
(97) Participant does not know

g. Coronary Heart Disease |___|___|
(00) No
(01) Yes
(99) Not documented
(98) Prefers not to answer
(97) Participant does not know

41. Which of the following NCDs do you currently suffer from?

a. Diabetes ("High Sugar") |___|___|
(00) No
(01) Yes
(99) Not documented
(98) Prefers not to answer
(97) Participant does not know

i. If yes, please indicate date of diagnosis
|___|___| / |___|___|/ |___|___||___|___|
DD/MM/YYYY

If day, month, or year are not known or not documented write"99"/"99"/"9999" as required

ii. Are you currently taking any medication for this NCD? |___|___|
(00) No
(01) Yes
(99) Not documented
(98) Prefers not to answer
(97) Participant does not know

If No, go to question 41b.

iii. If yes, please specify ***all*** medication(s) being taken: |___|___| |___|___| |___|___|
(00) NONE

(01) Acarbose (Precose)
(02) Chlorpropamine (Biabinese)
(03) Glimepiride (Amaryl)
(04) Glipizide (Glucotrol)
(05) Glyburide (DiaBeta/Glynase/Mictronase/Glucovance)
(06) Metformin (Glucophage/Glucovance)
(07) Miglitol (Glyset)
(08) Nateglinide (Starlix)
(09) Pioglitazone (Actos)
(10) Repaglinide (Prandin)
(11) Rosiglitazone (Avandia)
(12) Sitagliptin (Juanvia)
(13) Tolazamide (Tolinase)
(14) Tolbutamide (Orinase)
(15) On medication type unknown
(88) Other regimen, specify:___________________

iv. Do you have a treatment history with subcutaneous Insulin? |___|___|
(00) No
(01) Yes
(99) Not documented
(98) Prefers not to answer
(97) Participant does not know

v. When did you start your diabetes medication (of any kind)?
|___|___| / |___|___|/ |___|___|___|___|
DD/MM/YYYY

vi. Source of diabetes medication information? |___|___|
(00) Self-report
(01) Pill bottles/ count
(02) EMR/ SmartCare review
(03) Other routine record review
(88) Other, please specify: _____________________________

vii. In the past 7 days, how many doses of diabetes medication has the participant missed?
|___|___|
(00) None
(01) One
(02) Two
(03) Three
(04) Four or more
(98) Prefers not to answer
(97) Participant does not know

viii. If the participant missed at least one dose, what was the reason? |___|___|

(00) Chose not to take the medication due to side effects.
(01) Chose not to take the medication because couldn't take in private.
(02) Missed dose because participant had run out of medication.
(03) Missed dose because participant forgot to carry the medication.
(98) Prefers not to answer
(97) Participant does not know
(88) Other reason, please specify: ______________________

ix. In the last six months, did you miss the collection of your diabetes medication at this or another facility?
(00) No
(01) Yes
(98) Prefers not to answer
(97) Participant does not know
If No, skip to question x.

a. If yes, did you buy the diabetes medications from a private pharmacy during this time?
(00) No
(01) Yes
(98) Prefers not to answer
(97) Participant does not know
If No, skip to question x.

b. If yes, please tell us when and where you bought medications from a private pharmacy?

Date of Visit	Name of Private Pharmacy		Number of days Collected
1. |___|___| / |___|___|/ |___|___|___|___| DD/MM/YYYY	(00) Link Pharmacy<br>(01) Cairo Pharmacy<br>(02) Medlink Pharmacy<br>(03) Jubilee Chemist<br>(04) Mega Pharmacy<br>(05) Local Pharmacy, name unknown<br>(88) Other, specify: ______	|___|___|	|___|___|___|
2. |___|___| / |___|___|/ |___|___|___|___| DD/MM/YYYY		|___|___|	|___|___|___|
3. |___|___| / |___|___|/ |___|___|___|___| DD/MM/YYYY		|___|___|	|___|___|___|
4. |___|___| / |___|___|/ |___|___|___|___| DD/MM/YYYY		|___|___|	|___|___|___|

x. In the last 6 months, did you stop taking your diabetes medications for more than 7 days at any time for any reason (i.e., "stopped" means that they did not resume taking the diabetes medications)? |___|___|
(00) No
(01) Yes
(98) Prefers not to answer
(97) Participant does not know

a. If "Yes", How long did you stay without taking your diabetes medication? |___|___|
(00) 0 – 7 days
(01) More than 7 days but less than 30 days

(02) One month (31-59 days)
(03) Two months (60-89 days)
(04) Three months (90-119 days)
(05) Four or more months (≥120 days)
(88) Other, specify
(98) Prefers not to answer
(97) Participant does not know

b. If you stopped medication for more than 7 days, what was the reason? |___|___|

(00) Side effects.
(01) Advised to stop by a clinician.
(02) Medication was not available at the facility.
(03) Could not afford to buy medication.
(04) Started alternative medicine/myths in place of medication (i.e., water therapy)
(05) Taking traditional herbs/medication
(06) I was tired of taking the medication.
(07) I lost my supply of medication.
(88) Other, specify: _______________
(98) Prefers not to answer

xi. Do you ever forget to take your diabetes medication? |___|___|

(00) No
(01) Yes
(98) Prefers not to answer
(97) Participant does not know

xii. Are you negligent/ forgetful at times about taking your diabetes medication? |___|___|

(00) No
(01) Yes
(98) Prefers not to answer
(97) Participant does not know

xiii. When you feel better, do you sometimes stop taking your diabetes medication? |___|___|

(00) No
(01) Yes
(98) Prefers not to answer
(97) Participant does not know

xiv. Sometimes if you feel worse when you take the diabetes medication, do you stop taking it? |___|___|

(00) No
(01) Yes
(98) Prefers not to answer
(97) Participant does not know

xv. You only take your diabetes medication only when I feel sick? |___|___|

(00) No
(01) Yes
(98) Prefers not to answer
(97) Participant does not know

xvi. Do you think it is unnatural for your mind and body to be controlled by your diabetes. |___|___|

(00) No
(01) Yes
(98) Prefers not to answer
(97) Participant does not know

xvii. Your thoughts are clearer on your diabetes medication? |___|___|

(00) No
(01) Yes
(98) Prefers not to answer
(97) Participant does not know

xviii. By staying on your diabetes medication, you can prevent getting sick? |___|___|

(00) No
(01) Yes
(98) Prefers not to answer
(97) Participant does not know

xix. Diabetes medication makes you feel tired and sluggish. |___|___|

(00) No
(01) Yes
(98) Prefers not to answer
(97) Participant does not know

b. Hypertension (High “BP”) |___|___|

(00) No
(01) Yes
(99) Not documented
(98) Prefers not to answer
(97) Participant does not know

i. If yes, please indicate date of diagnosis |___|___| / |___|___|/ |___|___|___|___|
DD/MM/YYYY

If day, month, or year are not known or not documented write"99"/"99"/"9999" as required

ii. Are you currently taking any medication for this condition (hypertension)? |___|___|

(00) No
(01) Yes
(99) Not documented
(98) Prefers not to answer
(97) Participant does not know

If No, go to question 41c

iii. If yes, please specify ***all*** medication(s) being taken: |___|___| |___|___| |___|___|

(00) NONE
(01) Atenolol (Tenormin/Tenoretic)
(02) Bisoprolol (Zebeta/Ziac)
(03) Carvedilol (Coreg)
(04) Labetalol (Normodyne; Trandate)
(05) Metoprolol (Lopressor; Toprol)
(06) Nadolol (Corgard/Corzide)
(07) Pindolol (Visken)
(08) Propranolol (Inderal/Inderide)
(09) Timolol (Blocadren/Timolide)
(10) Benazepril (Lotensin/Lotrel)
(11) Captopril (Capoten/Capozide)
(12) Enalapril (Vasotec/Lexxel/Teczem/Vasretic)
(13) Fosinopril (Monopril)
(14) Lisinopril (Prinivil/Zestril/Prinzide/Zestoretic)
(15) Moexipril (Univasc/Uniretic)
(16) Quinapril (Accupril/Accuretic)
(17) Ramipril (Altace)
(18) Candesartan (Atacand)
(19) Irbesartan (Avapro/Avalide)
(20) Losartan (Cozaar/Hyzaar)
(21) Olmesartan (Benicar)
(22) Telmisartan (Micardis)
(23) Valsartan (Diovan)
(24) Amlodipine (Norvasc; Lotrel)
(25) Diltiazem (Cardizem/Cartia/Dilacor/Tiazac)
(26) Felodipine (Plendil)
(27) Nicardipine (Cardene)
(28) Nifedipine (Adalat/Procardia)
(29) Verapamil (Calan/Covera/Isoptin/Verelan)
(30) Clonidine (Catapres)

(31) Doxazosin (Cardura/)
(32) Methyldopa (Aldomet)
(33) Prazosin (Minipress)
(34) Terazosin (Hytrin)
(35) Digoxin
(36) Isosorbide Dinitrate (Dilatrate/Isordil/sorbitrate/BiDil)
(37) Isosorbide Mononitrate (Imdur/ISMO/Monoket)
(38) Bumetanide (Bumex)
(39) Furosemide (Lasix)
(40) Torsemide (Demadex)
(41) Chlorothiazide (Diuril)
(42) Chlorthalidone (Hygroton)
(43) Hydrochlorothiazide (Esidrix/HydroDiuril/Micozide/Oretic/Aldactazide/Moduretic/Spironazide/Zestoretic)
(44) Hydrochlorothiazide/Triamterene (Dyazide/Maxzide)
(45) Indapamide (Lozol)
(46) Metolazone (Microzide/Zaroxolyn)
(47) Acetazolamide (Diamox)
(48) Amiloride (Midamor/Moduretic)
(49) Spironolactone (Aldactone/Aldactazide/Spironaide/Spirozide)
(50) Triamterene (Dyrenium/Dyazide)
(51) Eplerenone (Inspra)
(52) Diazoxide (Hyperstat)
(53) Hydralazine (Apresoline/Apresazide/BiDil)
(54) On medication, type unknown
(88) Other regimen, specify: ___________________

iv. When did you start taking your anti-hypertensive medications?
|___|___| / |___|___|/ |___|___|___|___|
DD/MM/YYYY

v. Source of hypertension medication information?
|___|___|
(00) Self-report
(01) Pill bottles/ count
(02) EMR/ SmartCare review
(03) Other routine record review
(88) Other, please specify: ______________________________

vi. In the past 7 days, how many doses of anti-hypertension medication has the participant missed?
|___|___|
(00) None
(01) One
(02) Two
(03) Three
(04) Four or more

(98) Prefers not to answer
(97) Participant does not know

vii. If the participant missed at least one dose, what was the reason?

|___|___|

(00) Chose not to take the medication due to side effects.
(01) Chose not to take the medication because couldn't take in private.
(02) Missed dose because participant had run out of medication.
(03) Missed dose because participant forgot to carry the medication.
(98) Prefers not to answer
(97) Participant does not know.
1. Other reason, please specify: _________________________________

viii. In the last 6 months, did you miss the collection of your anti-hypertensive medications at this or another facility?
(00) No
(01) Yes
(98) Prefers not to answer
(97) Participant does not know
If No, skip to question ix.

a. If yes, did you buy your anti-hypertensive medications from a private pharmacy during this time?
(00) No
(01) Yes
(98) Prefers not to answer
(97) Participant does not know
If No, skip to question ix.

b. If yes, please tell us when and where you bought medications from a private pharmacy.

**Date of Visit**	**Name of Private Pharmacy**		**Number of days**
1. |___|___| / |___|___|/ |___|___|___|___| DD/MM/YYYY	(00) Link Pharmacy<br>(01) Cairo Pharmacy<br>(02) Medlink Pharmacy<br>(03) Jubilee Chemist<br>(04) Mega Pharmacy<br>(05) Local Pharmacy, name unknown<br>(88) Other, specify: ______	|___|___|	|___|___|___|
2. |___|___| / |___|___|/ |___|___|___|___| DD/MM/YYYY		|___|___|	|___|___|___|
3. |___|___| / |___|___|/ |___|___|___|___| DD/MM/YYYY		|___|___|	|___|___|___|
4. |___|___| / |___|___|/ |___|___|___|___| DD/MM/YYYY		|___|___|	|___|___|___|

ix. In the last 6 months, did you stop taking your anti-hypertensive medications for more than 7 days at any time for any reason (i.e., "stopped" means that they did not resume taking anti-hypertensive medications)?

|___|___|
(00) No
(01) Yes

(98) Prefers not to answer
(97) Participant does not know

x. If "Yes", How long did you stay without taking your anti-hypertensive medication? |___|___|

(00) 0 – 7 days
(01) More than 7 days but less than 30 days
(02) One month (31-59 days)
(03) Two months (60-89 days)
(04) Three months (90-119 days)
(05) Four or more months (≥120 days)
(88) Other, specify
(98) Prefers not to answer
(97) Participant does not know

xi. If you stopped medication for more than 7 days, what was the reason?

|___|___|

(00) Side effects.
(01) Advised to stop by clinician.
(02) Medication was not available at the facility.
(03) Could not afford to buy medication.
(04) Started alternative medicine/myths in place of medication (i.e., water therapy)
(05) Taking traditional herbs/medication
(06) I was tired of taking the medication.
(07) I lost my supply of medication.
(88) Other, specify: _______________
(98) Prefers not to answer

xii. Do you ever forget to take your anti-hypertensive medication? |___|___|

(00) No
(01) Yes
(98) Prefers not to answer
(97) Participant does not know

xiii. Are you negligent/ forgetful at times about taking your anti-hypertensive medication? |___|___|

(00) No
(01) Yes
(98) Prefers not to answer
(97) Participant does not know

xiv. When you feel better, do you sometimes stop taking your anti - hypertensive medication? |___|___|

(00) No
(01) Yes
(98) Prefers not to answer
(97) Participant does not know

xv. Sometimes if you feel worse when you take the anti - hypertensive medication, do you stop taking it?

|___|___|

(00) No
(01) Yes
(98) Prefers not to answer
(97) Participant does not know

xvi. You only take your anti-hypertensive medication only when I feel sick?
|___|___|

(00) No
(01) Yes
(98) Prefers not to answer
(97) Participant does not know

xvii. Do you think it is unnatural for your mind and body to be controlled by your anti-hypertensive medication.

|___|___|

(00) No
(01) Yes
(98) Prefers not to answer
(97) Participant does not know

xviii. Your thoughts are clearer on your anti-hypertensive medication? |___|___|

(00) No
(01) Yes
(98) Prefers not to answer
(97) Participant does not know

xix. By staying on your anti – hypertensive medication, you can prevent getting sick?
|___|___|

(00) No
(01) Yes
(98) Prefers not to answer
(97) Participant does not know

xx. Anti-hypertensive medication makes you feel tired and sluggish.
|___|___|

(00) No
(01) Yes
(98) Prefers not to answer
(97) Participant does not know

c. Dyslipidemia (“High cholesterol”)
|___|___|

(00) No
(01) Yes
(99) Not documented
(98) Prefers not to answer
(97) Participant does not know

i. If yes, please indicate date of diagnosis |___|___| / |___|___|/ |___|___|___|___|
DD/MM/YYYY

If day, month, or year are not known or not documented write"99"/"99"/"9999" as required

ii. Are you currently taking any medication for this condition (dyslipidemia/ "high cholesterol")? |___|___|
(00) No
(01) Yes
(99) Not documented
(98) Prefers not to answer
(97) Participant does not know

If No, go to question 41d

iii. If yes, please specify ***all*** medication(s) being taken: |___|___| |___|___| |___|___|
(00) NONE
(01) Atorvastatin (Lipitor)
(02) Fluvastatin (Lescol)
(03) Lovastatin (Mevacor/Advicor)
(04) Pravastatin (Pravachol)
(05) Rosuvistatin (Crestor)
(06) Simvastatin (Zocor/Vytorin)
(07) Fenofibrate (Antara/Lipofen/Lofibra/Tricor/Triglide)
(08) Gemfibrozil (Lopid)
(09) Niacin (Niaspan/Slo-Niacin/Advicor)
(10) Cholestyramine (Questran)
(11) Colestipol (Colestid)
(12) Ezetimibe (Vytorin/Zetia)
(13) Fish Oil &Omega-3 (Omacor)
(14) On medication, type unknown
(88) Other regimen, specify: ___________________

iv. When did you start taking your lipid/cholesterol lowering medication?
|___|___| / |___|___|/ |___|___|___|___|
DD/MM/YYYY

v. Source of lipid/ cholesterol lowering medication information.
|___|___|
(00) Self-report
(01) Pill bottles/ count
(02) EMR/ SmartCare review

(03) Other routine record review
(88) Other, please specify: ______________________________

vi. In the past 7 days, how many doses of lipid/ cholesterol lowering medication has the participant missed? |___|___|
(00) None
(01) One
(02) Two
(03) Three
(04) Four or more
(98) Prefers not to answer
(97) Participant does not know

vii. If the participant missed at least one dose, what was the reason? |___|___|
(00) Chose not to take the medication due to side effects.
(01) Chose not to take the medication because couldn't take in private.
(02) Missed dose because participant had run out of medication.
(03) Missed dose because participant forgot to carry the medication.
(98) Prefers not to answer
(97) Participant does not know
(88) Other reason, please specify: _________________

viii. In the last three months, did you miss the collection of your lipid profile lowering medication at this or another facility?
(00) No
(01) Yes
(98) Prefers not to answer
(97) Participant does not know
If No, skip to question ix.

a. If yes, did you buy the lipid profile lowering from a private pharmacy during this time?
(00) No
(01) Yes
(98) Prefers not to answer
(97) Participant does not know
If No, skip to question ix.

b. If yes, please tell us when and where you bought medications from a private pharmacy?

**Date of Visit**	**Name of Private Pharmacy**		**Number of days**
1. |___|___| / |___|___|/ |___|___|___|___| DD/MM/YYYY	(00) Link Pharmacy<br>(01) Cairo Pharmacy<br>(02) Medlink Pharmacy<br>(03) Jubilee Chemist	|___|___|	|___|___|___|
2. |___|___| / |___|___|/ |___|___|___|___| DD/MM/YYYY		|___|___|	|___|___|___|

3. |___|___| / |___|___|/ |___|___|___|___| DD/MM/YYYY	(04) Mega Pharmacy<br>(05) Local Pharmacy, name unknown<br>(88) Other, specify: ______	|___|___|	|___|___|___|
4. |___|___| / |___|___|/ |___|___|___|___| DD/MM/YYYY		|___|___|	|___|___|___|

ix. In the last 6 months, did you stop taking your lipid lowering medications for more than 7 days at any time for any reason (i.e., "stopped" means that they did not resume taking the lipid lowering medications)? |___|___|
- (00) No
- (01) Yes
- (98) Prefers not to answer
- (97) Participant does not know

x. If "Yes", How long did you stay without taking your lipid/cholesterol-lowering medication? |___|___|
- (00) 0 – 7 days
- (01) More than 7 days but less than 30 days
- (02) One month (31-59 days)
- (03) Two months (60-89 days)
- (04) Three months (90-119 days)
- (05) Four or more months (≥120 days)
- (88) Other, specify
- (98) Prefers not to answer
- (97) Participant does not know

xi. If you stopped medication for more than 7 days, what was the reason? |___|___|
- (00) Side effects.
- (01) Advised to stop by clinician.
- (02) Medication was not available at the facility.
- (03) Could not afford to buy medication.
- (04) Started alternative medicine/myths in place of medication (i.e., water therapy)
- (05) Taking traditional herbs/medication
- (06) I was tired of taking the medication.
- (07) I lost my supply of medication.
- (88) Other reason, specify: ________________
- (98) Prefers not to answer

xii. Do you ever forget to take your lipid lowering medication medication? |___|___|
- (00) No
- (01) Yes
- (98) Prefers not to answer
- (97) Participant does not know

xiii. Are you negligent/ forgetful at times about taking your lipid lowering medication? |___|___|
- (00) No
- (01) Yes

(98) Prefers not to answer
(97) Participant does not know

xiv. When you feel better, do you sometimes stop taking your lipid lowering medication? |___|___|

(00) No
(01) Yes
(98) Prefers not to answer
(97) Participant does not know

xv. Sometimes if you feel worse when you take the lipid lowering medication, do you stop taking it? |___|___|

(00) No
(01) Yes
(98) Prefers not to answer
(97) Participant does not know

xvi. You only take your lipid lowering medication only when I feel sick? |___|___|

(00) No
(01) Yes
(98) Prefers not to answer
(97) Participant does not know

xvii. Do you think it is unnatural for your mind and body to be controlled by your lipid lowering medication. |___|___|

(00) No
(01) Yes
(98) Prefers not to answer
(97) Participant does not know

xviii. Your thoughts are clearer on your lipid lowering medication? |___|___|

(00) No
(01) Yes
(98) Prefers not to answer
(97) Participant does not know

xix. By staying on your lipid lowering medication, you can prevent getting sick? |___|___|

(00) No
(01) Yes
(98) Prefers not to answer
(97) Participant does not know

xx. Lipid lowering medication makes you feel tired and sluggish. |___|___|
(00) No
(01) Yes
(98) Prefers not to answer
(97) Participant does not know

d. Other cardio metabolic NCDs (e.g., stroke, heart failure, etc.)? |___|___|
(00) No
(01) Yes
(88) Other, please specify
(99) Not documented
(98) Prefers not to answer
(97) Participant does not know

i. If yes, please indicate the name of the condition______________________________________

ii. If yes, please indicate date of diagnosis |___|___| / |___|___|/ |___|___|___|___|
DD/MM/YYYY

If day, month, or year are not known or not documented write"99"/"99"/"9999" as required

iii. Are you currently taking any medication for this NCD? |___|___|
(00) No
(01) Yes
(99) Not documented
(98) Prefers not to answer
(97) Participant does not know

If No, go to question 42

iv. If Yes, please specify ***all*** medication(s) being taken:
____
____
____
____

v. When did you start taking your other NCD medication?
|___|___| / |___|___|/ |___|___|___|___|
DD/MM/YYYY

vi. In the past 7 days, how many doses of medication for the other NCD has the participant missed?
|___|___|
(00) None
(01) One
(02) Two
(03) Three

(04) Four or more
(98) Prefers not to answer
(97) Participant does not know

vii. If participant missed at least one dose, what was the reason? |___|___|

(00) Chose not to take the medication due to side effects.
(01) Chose not to take the medication because couldn't take in private.
(02) Missed dose because participant had run out of medication.
(03) Missed dose because participant forgot to carry the medication
(98) Prefers not to answer
(97) Participant does not know.
(88) Other reason, please specify:______________

viii. In the last 6 months, did you miss the collection of your other NCD(s) medications at this or another facility?

(00) No
(01) Yes
(98) Prefers not to answer
(97) Participant does not know

If No, skip to question ix.

a. If yes, did you buy your other NCD(s) medications from a private pharmacy during this time?

(00) No
(01) Yes
(98) Prefers not to answer
(97) Participant does not know

If No, skip to question ix.

b. If yes, please tell us when and where you bought medications from a private pharmacy?

**Date of Visit**	**Name of Private Pharmacy**		**Number of days**
1. |___|___| / |___|___|/ |___|___|___|___| DD/MM/YYYY	(00) Link Pharmacy<br>(01) Cairo Pharmacy<br>(02) Medlink Pharmacy<br>(03) Jubilee Chemist<br>(04) Mega Pharmacy<br>(05) Local Pharmacy, name unknown<br>(88) Other, specify: ______	|___|___|	|___|___|___|
2. |___|___| / |___|___|/ |___|___|___|___| DD/MM/YYYY		|___|___|	|___|___|___|
3. |___|___| / |___|___|/ |___|___|___|___| DD/MM/YYYY		|___|___|	|___|___|___|
4. |___|___| / |___|___|/ |___|___|___|___| DD/MM/YYYY		|___|___|	|___|___|___|

ix. In the last 6 months, did you stop taking the medication for more than one day at any time for any reason (i.e., "stopped" means that they did not resume taking the medication)? |___|___|

(00) No
(01) Yes
(98) Prefers not to answer
(97) Participant does not know

x. If "Yes", How long did you stay without taking your medication for other NCDs? |___|___|

(00) 0 – 7 days
(01) More than 7 days but less than 30 days
(02) One month (31-59 days)
(03) Two months (60-89 days)
(04) Three months (90-119 days)
(05) Four or more months (≥120 days)
(88) Other, specify
(98) Prefers not to answer
(97) Participant does not know

xi. If you stopped medication for more than 7 days, what was the reason?

|___|___|

(00) Side effects.
(01) Advised to stop by clinician.
(02) Medication was not available at the facility.
(03) Could not afford to buy medication.
(04) Started alternative medicine/myths in place of medication (i.e., water therapy)
(05) Taking traditional herbs/medication
(06) I was tired of taking the medication
(07) I lost my supply of medication.
(88) Other reason, specify: _______________
(98) Prefers not to answer

xii. Do you ever forget to take your other NCD(s) medication?
|___|___|

(00) No
(01) Yes
(98) Prefers not to answer
(97) Participant does not know

xiii. Are you negligent/ forgetful at times about taking your other NCD(s) medication? |___|___|

(00) No
(01) Yes
(98) Prefers not to answer
(97) Participant does not know

xiv. When you feel better, do you sometimes stop taking your other NCD(s) medication?

|___|___|

(00) No
(01) Yes
(98) Prefers not to answer
(97) Participant does not know

xv. Sometimes if you feel worse when you take the other NCD(s) medication, do you stop taking it? |___|___|

(00) No
(01) Yes
(98) Prefers not to answer
(97) Participant does not know

xvi. You only take your other NCD(s) medication only when I feel sick? |___|___|

(00) No
(01) Yes
(98) Prefers not to answer
(97) Participant does not know

xvii. It is unnatural for your mind and body to be controlled by your other NCD(s) medication. |___|___|

(00) No
(01) Yes
(98) Prefers not to answer
(97) Participant does not know

xviii. Your thoughts are clearer on your other NCD(s) medication? |___|___|

(00) No
(01) Yes
(98) Prefers not to answer
(97) Participant does not know

xix. By staying on your NCD(s) medication, you can prevent getting sick? |___|___|

(00) No
(01) Yes
(98) Prefers not to answer
(97) Participant does not know

xx. Other NCD(s) medication makes you feel tired and sluggish. |___|___|

(00) No
(01) Yes
(98) Prefers not to answer
(97) Participant does not know

PART F: HISTORY OF CARDIOVASCULAR DISEASES

42. History of a myocardial infarction or heart attack |___|___|
    - (00) No
    - (01) Yes
    - (99) Not documented
    - (98) Prefers not to answer
    - (97) Participant does not know

43. History of stroke |___|___|
    - (00) No
    - (01) Yes
    - (99) Not documented
    - (98) Prefers not to answer
    - (97) Participant does not know

44. History of any form of heart arrhythmia |___|___|
    - (00) No
    - (01) Yes
    - (99) Not documented
    - (98) Prefers not to answer
    - (97) Participant does not know

45. History of other CVDs including Rheumatic heart disease, Peripheral arterial disease, etc. |___|___|
    - (00) No
    - (01) Yes
    - (99) Not documented
    - (98) Prefers not to answer
    - (97) Participant does not know

46. In the last 12 months have you been admitted/ hospitalized at a health facility? |___|___|
    - (00) No
    - (01) Yes
    - (99) Not documented
    - (98) Prefers not to answer
    - (97) Participant does not know

i. If your response is "Yes", please indicate when and where you were admitted / hospitalized and what the problem was for:
If day, month, or year are not known or not documented write"99"/"99"/"9999" as required.

Date of admission	Reason for admission (Include all applicable)		Name of health facility	
1. |___|___| / |___|___|/ |___|___||___|___| *DD/MM/YYYY*	(01) Hypertension<br>(02) Diabetes<br>(03) Chest Pain<br>(04) Heart Attack/Myocardial Infarction<br>(05) Stroke<br>(06) Heart Arrhythmias<br>(07) Other CVDs like rheumatic heart disease, peripheral arterial disease<br>(08) Kidney problems<br>(09) Liver problems<br>(10) Tuberculosis<br>(11) COVID-19<br>(12) Problems related to HIV<br>(88) Other, specify:<br>_____________________	|___|___| |___|___| |___|___|	(01) Bauleni Urban Health Centre<br>(02) Kanyama 1st Level Hospital<br>(03) Chipata 1st Level Hospital<br>(04) Chawama 1st Level Hospital<br>(05) Chelstone Urban Health Centre<br>(06) Kalingalinga Urban Health Centre<br>(07) Kamwala Urban Health Centre<br>(08) Makeni Urban Health Centre<br>(09) M'tendere Urban Health Centre<br>(10) Railway Urban Health Centre<br>(11) Kabwata Urban Health Centre<br>(12) N'gombe Urban Health Centre<br>(13) Chilenje 1st Level Hospital<br>(14) George Urban Health Centre<br>(15) Matero Ref 1st Level Hospital<br>(16) UTH<br>(17) Levy<br>(18) Maina Soko<br>(88) Other, specify:_____________	|___|___|
2. |___|___| / |___|___|/ |___|___||___|___| *DD/MM/YYYY*		|___|___| |___|___| |___|___|		|___|___|
3. |___|___| / |___|___|/ |___|___||___|___| *DD/MM/YYYY*		|___|___| |___|___| |___|___|		|___|___|
4. |___|___| / |___|___|/ |___|___||___|___| *DD/MM/YYYY*		|___|___| |___|___| |___|___|		|___|___|
5. |___|___| / |___|___|/ |___|___||___|___| *DD/MM/YYYY*		|___|___| |___|___| |___|___|		|___|___|

47. Please tell us if you came to this or another clinic in the last 12 months for a clinical review, laboratory testing, or to pick up medications for any of the following conditions, and, if you did, when did you visit?

If day, month, or year are not known or not documented write"99"/"99"/"9999" as required.

a. HIV |___|___|

(00) No
(01) Yes
(99) Not documented
(98) Prefers not to answer
(97) Participant does not know

If "Yes" skip to ii.

i. If "No"/ "Not documented" / "Prefers not to answer" / "Participant does not know," did you go to a private clinic/ hospital?

(00) No
(01) Yes
(99) Not documented
(98) Prefers not to answer
(97) Participant does not know

TASKPEN UH3 Patient Survey v1.1

|___|___|___|-|___|___|___|___| |___|___| / |___|___|/ |___|___|___|___|
Site Code - Participant ID DD/MM/YYYY

Date of Visit	Reason for Visit (Include all that apply)		Name of health facility	
1. |___|___| / |___|___|/ |___|___|___|___| DD/MM/YYYY	(00) Clinical Review<br>(01) Laboratory tests<br>(02) Medication pick up<br>(88) Other, Specify	|___|___| |___|___| |___|___|	(00) Bauleni Urban Health Centre<br>(01) Kanyama 1st Level Hospital<br>(02) Chipata 1st Level Hospital<br>(03) Chawama 1st Level Hospital<br>(04) Chelstone Urban Health Centre<br>(05) Kalingalinga Urban Health Centre<br>(06) Kamwala Urban Health Centre<br>(07) Makeni Urban Health Centre<br>(08) M'tendere Urban Health Centre<br>(09) Railway Urban Health Centre<br>(10) Kabwata Urban Health Centre<br>(11) N'gombe Urban Health Centre<br>(12) Chilenje 1st Level Hospital<br>(13) George Urban Health Centre<br>(14) Matero Ref 1st Level Hospital<br>(88) Other, specify<br>______________________________	|___|___|
2. |___|___| / |___|___|/ |___|___|___|___| DD/MM/YYYY		|___|___| |___|___| |___|___|		|___|___|
3. |___|___| / |___|___|/ |___|___|___|___| DD/MM/YYYY		|___|___| |___|___| |___|___|		|___|___|
4. |___|___| / |___|___|/ |___|___|___|___| DD/MM/YYYY		|___|___| |___|___| |___|___|		|___|___|

b. Hypertension (High "BP") |___|___|
(00) No
(01) Yes
(99) Not documented
(98) Prefers not to answer
(97) Participant does not know

If "Yes" skip to ii.

i. If "No"/ "Not documented" / "Prefers not to answer" / "Participant does not know," did you go to a private clinic/ hospital? |___|___|

(00) No
(01) Yes
(99) Not documented
(98) Prefers not to answer
(97) Participant does not know

TASKPEN UH3 Patient Survey v1.1

|___|___|___|-|___|___|___|___| |___|___| / |___|___|/ |___|___|___|___|
Site Code - Participant ID DD/MM/YYYY

Date of Visit	Reason for Visit (Include all that apply)		Name of health facility	
1. |___|___| / |___|___|/ |___|___|___|___| *DD/MM/YYYY*	(00) Clinical Review<br>(01) Laboratory tests<br>(02) Medication pick up<br>(88) Other, Specify	|___|___| |___|___| |___|___|	(00) Bauleni Urban Health Centre<br>(01) Kanyama 1st Level Hospital<br>(02) Chipata 1st Level Hospital<br>(03) Chawama 1st Level Hospital<br>(04) Chelstone Urban Health Centre<br>(05) Kalingalinga Urban Health Centre<br>(06) Kamwala Urban Health Centre<br>(07) Makeni Urban Health Centre<br>(08) M'tendere Urban Health Centre<br>(09) Railway Urban Health Centre<br>(10) Kabwata Urban Health Centre<br>(11) N'gombe Urban Health Centre<br>(12) Chilenje 1st Level Hospital<br>(13) George Urban Health Centre<br>(14) Matero Ref 1st Level Hospital<br>(88) Other, specify<br>______________________________	|___|___|
2. |___|___| / |___|___|/ |___|___|___|___| *DD/MM/YYYY*		|___|___| |___|___| |___|___|		|___|___|
3. |___|___| / |___|___|/ |___|___|___|___| *DD/MM/YYYY*		|___|___| |___|___| |___|___|		|___|___|
4. |___|___| / |___|___|/ |___|___|___|___| *DD/MM/YYYY*		|___|___| |___|___| |___|___|		|___|___|

c. Diabetes |___|___|

(00) No
(01) Yes
(99) Not documented
(98) Prefers not to answer
(97) Participant does not know

If "Yes" skip to ii.

i. If "No"/ "Not documented" / "Prefers not to answer" / "Participant does not know," did you go to a private clinic/ hospital?
   - (00) No
   - (01) Yes
   - (99) Not documented
   - (98) Prefers not to answer
   - (97) Participant does not know

**Date of Visit**	**Reason for Visit (Include all that apply)**		**Name of health facility**	
1. |___|___| / |___|___|/ |___|___|___|___| *DD/MM/YYYY*	(00) Clinical Review<br>(01) Laboratory tests<br>(02) Medication pick up<br>(88) Other, Specify	|___|___| |___|___| |___|___|	(00) Bauleni Urban Health Centre<br>(01) Kanyama 1st Level Hospital<br>(02) Chipata 1st Level Hospital<br>(03) Chawama 1st Level Hospital<br>(04) Chelstone Urban Health Centre<br>(05) Kalingalinga Urban Health Centre<br>(06) Kamwala Urban Health Centre<br>(07) Makeni Urban Health Centre<br>(08) M'tendere Urban Health Centre<br>(09) Railway Urban Health Centre<br>(10) Kabwata Urban Health Centre<br>(11) N'gombe Urban Health Centre<br>(12) Chilenje 1st Level Hospital<br>(13) George Urban Health Centre<br>(14) Matero Ref 1st Level Hospital<br>(88) Other, specify<br>_______________________	|___|___|
2. |___|___| / |___|___|/ |___|___|___|___| *DD/MM/YYYY*		|___|___| |___|___| |___|___|		|___|___|
3. |___|___| / |___|___|/ |___|___|___|___| *DD/MM/YYYY*		|___|___| |___|___| |___|___|		|___|___|
4. |___|___| / |___|___|/ |___|___|___|___| *DD/MM/YYYY*		|___|___| |___|___| |___|___|		|___|___|

d. Dyslipidemia ("High Cholesterol") |___|___|

(00) No
(01) Yes
(99) Not documented
(98) Prefers not to answer
(97) Participant does not know

If "Yes" skip to ii.

i. If "No"/ "Not documented" / "Prefers not to answer" / "Participant does not know," did you go to a private clinic/ hospital?
   (00) No
   (01) Yes
   (99) Not documented
   (98) Prefers not to answer
   (97) Participant does not know

TASKPEN UH3 Patient Survey v1.1

|___|___|___|-|___|___|___|___| |___|___| / |___|___|/ |___|___|___|___|
Site Code - Participant ID DD/MM/YYYY

Date of Visit	Reason for Visit (Include all that apply)		Name of health facility	
1. |___|___| / |___|___|/ |___|___|___|___| DD/MM/YYYY	(00) Clinical Review<br>(01) Laboratory tests<br>(02) Medication pick up<br>(88) Other, Specify	|___|___| |___|___| |___|___|	(00) Bauleni Urban Health Centre<br>(01) Kanyama 1st Level Hospital<br>(02) Chipata 1st Level Hospital<br>(03) Chawama 1st Level Hospital<br>(04) Chelstone Urban Health Centre<br>(05) Kalingalinga Urban Health Centre<br>(06) Kamwala Urban Health Centre<br>(07) Makeni Urban Health Centre<br>(08) M'tendere Urban Health Centre<br>(09) Railway Urban Health Centre<br>(10) Kabwata Urban Health Centre<br>(11) N'gombe Urban Health Centre<br>(12) Chilenje 1st Level Hospital<br>(13) George Urban Health Centre<br>(14) Matero Ref 1st Level Hospital<br>(88) Other, specify<br>________________________	|___|___|
2. |___|___| / |___|___|/ |___|___|___|___| DD/MM/YYYY		|___|___| |___|___| |___|___|		|___|___|
3. |___|___| / |___|___|/ |___|___|___|___| DD/MM/YYYY		|___|___| |___|___| |___|___|		|___|___|
4. |___|___| / |___|___|/ |___|___|___|___| DD/MM/YYYY		|___|___| |___|___| |___|___|		|___|___|

e. Other condition, Specify______________________

(00) No
(01) Yes
(99) Not documented
(98) Prefers not to answer
(97) Participant does not know

If "Yes" skip to ii.

ii. If "No"/ "Not documented" / "Prefers not to answer" / "Participant does not know," did you go to a private clinic/ hospital?

(00) No
(01) Yes
(99) Not documented
(98) Prefers not to answer
(97) Participant does not know

TASKPEN UH3 Patient Survey v1.1

|___|___|___|-|___|___|___|___| |___|___| / |___|___|/ |___|___|___|___|
Site Code - Participant ID DD/MM/YYYY

Date of Visit	Reason for Visit (Include all that apply)		Name of health facility	
1. |___|___| / |___|___|/ |___|___|___|___| DD/MM/YYYY	(00) Clinical Review<br>(01) Laboratory tests<br>(02) Medication pick up<br>(88) Other, Specify	|___|___| |___|___| |___|___|	(00) Bauleni Urban Health Centre<br>(01) Kanyama 1st Level Hospital<br>(02) Chipata 1st Level Hospital<br>(03) Chawama 1st Level Hospital<br>(04) Chelstone Urban Health Centre<br>(05) Kalingalinga Urban Health Centre<br>(06) Kamwala Urban Health Centre<br>(07) Makeni Urban Health Centre<br>(08) M'tendere Urban Health Centre<br>(09) Railway Urban Health Centre<br>(10) Kabwata Urban Health Centre<br>(11) N'gombe Urban Health Centre<br>(12) Chilenje 1st Level Hospital<br>(13) George Urban Health Centre<br>(14) Matero Ref 1st Level Hospital<br>(88) Other, specify ______________________	|___|___|
2. |___|___| / |___|___|/ |___|___|___|___| DD/MM/YYYY		|___|___| |___|___| |___|___|		|___|___|
3. |___|___| / |___|___|/ |___|___|___|___| DD/MM/YYYY		|___|___| |___|___| |___|___|		|___|___|
4. |___|___| / |___|___|/ |___|___|___|___| DD/MM/YYYY		|___|___| |___|___| |___|___|		|___|___|

PART I: NUTRITION

48. In a typical week, on how many days do you eat fruit? |___|___|

(00) None (Zero days)
(01) One day
(02) Two days
(03) Three days
(04) Four days
(05) Five days
(06) Six days
(07) Seven days
(98) Prefers not to answer
(97) Participant does not know

a. On average, how many servings of fruit do you eat on one of those days? |___|___|.|___|
Write 97 if the participant does not know

49. In a typical week, on how many days do you eat vegetables? |___|___|

(00) None (Zero days)
(01) One day
(02) Two days
(03) Three days
(04) Four days
(05) Five days
(06) Six days
(07) Seven days
(98) Prefers not to answer
(97) Participant does not know

a. On average, how many servings of vegetables do you eat on one of those days?
|___|___|.|___|
Write 97 if the participant does not know

50. In a typical week, on how many days do you eat fish (includes fresh and dry fish, large and small)?
|___|___|

(00) None (Zero days)
(01) One day
(02) Two days
(03) Three days
(04) Four days
(05) Five days
(06) Six days
(07) Seven days
(98) Prefers not to answer
(97) Participant does not know

b. On average, how many servings of fish do you eat on one of those days? |____|____|.|____|
Write 97 if the participant does not know

51. In a typical week, on how many days do you eat whole grains (like brown or wild rice, oatmeal, quinoa, barley, cracked wheat, or whole-wheat bread, pasta, or crackers)? |____|____|
(00) None (Zero days)
(01) One day
(02) Two days
(03) Three days
(04) Four days
(05) Five days
(06) Six days
(07) Seven days
(98) Prefers not to answer
(97) Participant does not know

a. On average, how many servings of whole grains do you eat on one of those days? |____|____|.|____|

Write 97 if the participant does not know

52. In the last week, how often did you add salt or a salty sauce such as soya sauce to your food right before you ate it or as you were eating it? |____|____|

(00) Never
(01) Always (every meal)
(02) Often (majority meals)
(03) Sometimes (a couple of meals in a week)
(04) Rarely (once or twice a week)
(05) Very rarely (a couple of meals in a month)
(98) Prefers not to answer
(97) Participant does not know

53. How often is salt, salty seasoning or a salty sauce added in cooking or preparing foods in your household? |____|____|

(00) Never
(01) Always (every meal)
(02) Often (majority meals)
(03) Sometimes (a couple of meals in a week)
(04) Rarely (once or twice a week)
(05) Very rarely (a couple of meals in a month)
(98) Prefers not to answer
(97) Participant does not know

|___|___|___|-|___|___|___|___| Site Code - Participant ID

|___|___| / |___|___|/ |___|___|___|___| DD/MM/YYYY

54. How often do you eat processed food high in salt? By processed food high in salt, I mean foods that have been altered from their natural state, such as packaged salty snacks, canned salty food including pickles and preserves, salty food prepared at a fast-food restaurant, cheese, bacon, and processed meat such as polony, Hungarian sausages, biltong, salt-preserved fish, etc. |___|___|

(00) Never
(01) Always (every meal)
(02) Often (majority meals)
(03) Sometimes (a couple of meals in a week)
(04) Rarely (once or twice a week)
(05) Very rarely (a couple of meals in a month)
(98) Prefers not to answer
(97) Participant does not know

55. Do you think that too much salt or salty sauce in your diet could cause a health problem? |___|___|

(00) No
(01) Yes
(98) Prefers not to answer
(97) Participant does not know

56. Do you do any of the following regularly to control your salt intake?

a. Limit consumption of processed foods |___|___|
(00) No
(01) Yes
(98) Prefers not to answer
(97) Participant does not know

b. Look at the salt or sodium content on food labels |___|___|
(00) No
(01) Yes
(98) Prefers not to answer
(97) Participant does not know

c. Buy low salt/sodium alternatives |___|___|
(00) No
(01) Yes
(98) Prefers not to answer
(97) Participant does not know

d. Use spices other than salt when cooking |___|___|
(00) No
(01) Yes
(98) Prefers not to answer

(97) Participant does not know

e. Avoid eating foods prepared outside of a home |___|___|
(00) No
(01) Yes
(98) Prefers not to answer
(97) Participant does not know

f. Do other things specifically to control your salt intake |___|___|
(00) No
(01) Yes
(98) Prefers not to answer
(97) Participant does not know

i. If yes to, please specify: _______________________________

57. The next questions ask about the oil or fat that is most often used for meal preparation in your household, and about sugar that you eat.

a. What type of oil or fat is most often used for meal preparation in your household? |___|___|
(00) Vegetable/corn/olive/sunflower oil
(01) Lard or suet
(02) Butter
(03) Margarine
(04) None in particular
(05) None used
(88) Other, please specify: ____________________
(98) Prefers not to answer
(97) Participant does not know

In a typical week on how many days do you take sugary drinks or soda (carbonated drinks) like Fanta, Coca Cola, 7-Up, PEPSI, Merinda, etc.? |___|___|

(00) None (Zero days)
(01) One day
(02) Two days
(03) Three days
(04) Four days
(05) Five days
(06) Six days
(07) Seven days
(98) Prefers not to answer
(97) Participant does not know

b. On average, how many 300ml bottles do you take each time you drink sugary drinks or soda on one of those days?

|___|___|

Write 97 if the participant does not know

c. On a typical day, how many teaspoons of sugar do you add to your drinks and/or your food?

|___|___|

Write 97 if the participant does not know

PART J: PHYSICAL ACTIVITY
Work Related Physical Activity

58. Does your work involve vigorous intensity activity that causes large increases in breathing or heart rate like carrying or lifting heavy loads, digging or construction work for at least 10 minutes continuously?
    - (00) No
    - (01) Yes
    - (98) Prefers not to answer
    - (97) Participant does not know

    If "No" skip to 59

a. In a typical week, on how many days do you do vigorous – intensity activities as part of your work?

|___|___|

- (00) None (Zero days)
- (01) One day
- (02) Two days
- (03) Three days
- (04) Four days
- (05) Five days
- (06) Six days
- (07) Seven days
- (98) Prefers not to answer
- (97) Participant does not know

b. How much time do you spend doing vigorous – intensity activities at work on a typical day?

|___|___|:|___|___|
HH:MM

59. Does your work involve moderate intensity activity that causes small increases in breathing or heart rate such as brisk walking or carrying light loads for at least 10 minutes continuously?
    - (00) No
    - (01) Yes
    - (98) Prefers not to answer
    - (97) Participant does not know

If "No" skip to 60

a. In a typical week, on how many days do you do moderate intensity activities at work on a typical day? |___|___|
   (00) None (Zero days)
   (01) One day
   (02) Two days
   (03) Three days
   (04) Four days
   (05) Five days
   (06) Six days
   (07) Seven days
   (98) Prefers not to answer
   (97) Participant does not know
b. How much time do you spend doing moderate intensity activities at work on a typical day?
   |___|___|:|___|___|
   HH:MM

Travel to and from places related physical activity

60. Do you walk or use a bicycle for at least 10 minutes continuously to get to and from places? |___|___|
   (00) No
   (01) Yes
   (98) Prefers not to answer
   (97) Participant does not know

If "No" skip to 61

a. In a typical week, on how many days do you walk or bicycle for at least 10 minutes continuously to get to and from places? |___|___|
   (00) None (Zero days)
   (01) One day
   (02) Two days
   (03) Three days
   (04) Four days
   (05) Five days
   (06) Six days
   (07) Seven days
   (98) Prefers not to answer
   (97) Participant does not know
b. How much time do you spend walking or bicycle for travel on a typical day?
   |___|___|:|___|___|
   HH:MM

Recreational activities related physical activity

61. Do you do any vigorous intensity sports, fitness or recreational (leisure) activities that cause large increases in breathing or heart rate like running or football, netball for at least 10 continuously?
   |___|___|

(00) No
(01) Yes
(98) Prefers not to answer
(97) Participant does not know

If "No" skip to 62

a. In a typical week, on how many days do you spend doing vigorous – intensity sports, fitness, or recreational activities or a typical day? |___|___|
   - (00) None (Zero days)
   - (01) One day
   - (02) Two days
   - (03) Three days
   - (04) Four days
   - (05) Five days
   - (06) Six days
   - (07) Seven days
   - (98) Prefers not to answer
   - (97) Participant does not know

b. How much time do you spend doing vigorous intensity sports, fitness, or recreational activities on a typical day?

|___|___|:|___|___|
HH:MM

62. Do you do any moderate intensity sports, fitness or recreational (leisure) activities that cause a small increase in breathing or heart rate such as brisk walking, cycling swimming, for at least 10 minutes continuously? |___|___|
   - (00) No
   - (01) Yes
   - (98) Prefers not to answer
   - (97) Participant does not know

If "No", end survey

a. In a typical week, on how many days do you do moderate – intensity sports, fitness or recreational (leisure) activities? |___|___|
   - (00) None (Zero days)
   - (01) One day
   - (02) Two days
   - (03) Three days
   - (04) Four days
   - (05) Five days
   - (06) Six days
   - (07) Seven days
   - (98) Prefers not to answer

(97) participant does not know

b. How much time do you spend doing moderate intensity sports, fitness or recreational (leisure) activities on a typical day?

|___|___|:|___|___|
HH:MM

STOP –FORM COMPLETE
