# Supplementary material for: Evaluating a multifaceted implementation strategy and package of evidence-based interventions based on WHO PEN for people living with HIV and cardiometabolic conditions in Lusaka, Zambia: protocol for the TASKPEN hybrid effectiveness-implementation stepped wedge cluster randomized trial
Source: Implement Sci Commun. 2024 Jun 6;5:61. doi: 10.1186/s43058-024-00601-z (PMC11155136; doi:10.1186/s43058-024-00601-z)
Supplement: Supplementary file 2 — Additional file 2. includes the informed consent form for the main trial survey. [file 43058_2024_601_MOESM2_ESM.pdf]

THE UNIVERSITY OF ZAMBIA
BIOMEDICAL RESEARCH ETHICS COMMITTEE

Telephone: 256067
Telegrams: UNZA, LUSAKA
Telex: UNZALU ZA 44370
Fax: + 260-1-250753
E-mail: unzarec@unza.zm

Ridgeway Campus
P.O. Box 50110
Lusaka, Zambia

Assurance No. FWA00000338
IRB00001131 of IOR G0000774

INFORMED CONSENT FORM FOR ADULT SURVEY PARTICIPANTS

Title of study: Z32201 - Effects of a package of evidence-based interventions and implementation strategies based on WHO PEN for people living with HIV and cardio-metabolic conditions in Lusaka, Zambia: A type II hybrid effectiveness-implementation stepped wedge trial (short title: "TASKPEN UH3")

Consent form version date: Version 1.1, dated 14 February 2023
Principal Investigators: Dr. Wilbroad Mutale, UNZA & CIDRZ, Dr. Michael Herce, UNC & CIDRZ
Co-Investigators: Dr. Samuel Bosomprah, CIDRZ, Dr. Felix Masiye, UNZA, Dr. Michael Vinikoor, CIDRZ and UAB, Dr. Jessie K. Edwards, UNC, Dr. Oliver Mweemba, UNZA, Dr Maurice Musheke, CIDRZ, Dr. Mmamulatelo Siame, UNZA, Chomba Mandyata, UNZA, Tulani Matenga, UNZA, and Chilambwe Mwila, CIDRZ

Funding Source and/or Sponsor: U.S. National Institutes of Health (NIH)

Study Contact telephone number: +260 967-780-284
Study Principal Investigator Email: Wilbroad.Mutale@cidrz.org

Concise Summary

This is a research study to identify the most effective ways for the Zambian health care system to care for people with non-communicable diseases (NCDs) who are also living with HIV. Participation will involve a one-time questionnaire about your health and lifestyle, and having a small amount of blood collected to test for NCDs. Your medical records may also be reviewed to collect some health information about you. You may also be invited to follow up with the study if you're found to have hypertension ("BP"), diabetes ("high sugar"), and/or high cholesterol through the survey. Benefits of participation could include improved health care, as the study team will work to ensure you are receiving all Ministry of Health-recommended tests. Risks include possible breach of confidentiality and the risks associated with blood collection. If you are interested in learning more about this study, please continue to read below.

What are some general things you should know about research studies?
You are being asked to take part in a research study. Joining the study is voluntary. You may refuse to join, or you may withdraw your consent to be in the study, for any reason. Research studies are designed to obtain new knowledge that may help other people in the future. You may not receive any direct benefit from being in the research study. There also may be risks to being in research studies. Deciding not to be in the study or leaving the study before it is done will not affect your relationship with the researchers or your health care provider. It will also not have any effect on the quality of care you receive at this health facility. If you have an illness, you do not have to be in the research study to receive health care.

Details about this study are discussed below. It is important that you understand this information so that you can make an informed choice about being in this research study. You will be offered a copy of this consent form. You should ask the researchers named above, or staff members assisting them, any questions you have about this study at any time.

What is the purpose of this study?
Despite the high and rising number of non-communicable diseases (NCDs) like high blood pressure ("BP"), diabetes ("high sugar"), and high cholesterol among people living with HIV in Zambia, there remains a lack of good information on how best to take care of NCDs alongside HIV in hospitals, clinics, and the community. This study will add new information on how best to take care of NCDs like "BP" and diabetes while also treating HIV for people living with HIV in Zambia.

For this study, we are working with the Ministry of Health to improve the quality of NCD care and treatment for people living with HIV in several clinics in Lusaka by: 1) training nurses and community health workers to work alongside doctors and clinicians to treat common NCDs according to recommendations from the World Health Organization; 2) improving the labs in the clinics to be able to identify NCDs and check how well patients are doing with their NCDs; 3) improving the national electronic medical record (i.e., SmartCare) to address common NCDs; 4) taking advantage of the resources available for HIV services to integrate NCD treatment and care into the same ART clinics; and 5) making medications to treat NCDs more accessible and efficient. While the study focuses on how to integrate this care package into Zambian clinics to manage NCDs affecting the heart and blood vessels in people living with HIV (called "cardio-metabolic" conditions), lessons learned from this study could apply to other NCDs.

For this part of the study, we would like to check you for "BP", high cholesterol, and diabetes/ "high sugar," and, if these conditions are present, see how well they are being controlled. We also want to ask you questions about your medical history, overall health, and lifestyle.

Where is this study being conducted?
This part of the study is being conducted in Lusaka at twelve (12) health facilities with ART clinics.

Are there any reasons you should not be in this study?
You should not be in this study if you do not have HIV infection, if you are coming to this site only once to get healthcare, or have previously done a survey with the study. You can participate in this study if you are 18 years of age or older, and voluntarily consent to join the study.

How many people will take part in this study?

For this part of the study, about 5,100 people living with HIV from 12 health facilities will take part. For all parts of the study, there will be about 5,400 people in total directly taking part in various study activities.

How long will your part of this study last?

If you agree to take part in the study, we will ask you to complete a survey. During the survey, we will collect health information about you by talking to you, taking blood samples from you, and reviewing your medical record. To complete all study procedures today will take about 1 and 1/2 hours. If we can't complete all the study procedures today, we may ask you to come back in the next few days to complete them.

What will happen if you take part in the study?

If you agree to take part in the study, we will collect information about your health. We will ask you questions about your HIV and other health conditions, any treatments or medications you take, what foods you eat, any habits you may have that could affect your health like smoking or drinking alcohol, how you prefer to receive services for HIV and NCDs, and other questions related to your HIV and any possible cardio-metabolic condition(s). If we learn from the survey that you have "BP," diabetes ("high sugar"), high cholesterol and/or other NCD, we will contact you after the survey to see if you would like to be followed by the study over the next 24 months or so. We will also use the routine data collection practices at your facility to get information about your health, this will involve reviewing your medical records from the facility registers and electronic record (SmartCare).

We will check your height, weight, and waste circumference. We will also check your vital signs like your pulse and blood pressure. Finally, we collect a blood sample to check to see if you have diabetes ("high sugar") or high cholesterol and to see how your HIV condition is being controlled. This usually involves collecting 1 or 2 tubes of blood (about 5 to 10 ml, or 2 to 3 tablespoons). If possible, we will use a government-approved machine in the clinic to do this testing known as "point-of-care" testing. In the case of a point-of-care test, the amount of blood collected will be less and will be around 2-3 drops of whole blood (less than 10 microL) per test. You will be given the results of any tests that are performed. We will also give the results to the clinic so that they can treat any NCD conditions we find through the study. The samples collected from you will only be used for the testing described in this form. The samples will not be used for other research now or in the future. The samples will not be sold or used for commercial profit.

What are the possible benefits of being in this study?

There are a few ways you may benefit from being in this study. You may benefit by being asked questions or having testing done that may reveal possible health problems, for which you will be referred for further assistance at this clinic or another health facility. You may also benefit from receiving basic education and counseling on HIV and cardio-metabolic conditions. You may benefit from having study staff available to you for counseling or answering your questions about HIV and cardio-metabolic conditions, which might otherwise be harder to access. Lastly, we will share your lab test results with the clinic to help keep your HIV and any cardio-metabolic condition under control.

There may also be some indirect benefits if you take part in this study. You will help us learn if a package of services recommended by the World Health Organization improves HIV and cardio-metabolic outcomes for people living with HIV in Zambia. This will help us and the Ministry of Health improve the care and treatment of cardio-metabolic conditions and other non-communicable diseases for people living with HIV and potentially the general public.

What are the possible risks or discomforts involved with being in this study?
Although the risk is low, it is possible that your personal information could become known to someone who should not have it. If that happened, you could face discrimination, stress, and embarrassment. However, the risk of this happening is very low since we will take several steps to prevent that from happening.

Some of the questions we ask about your health or habits could be sensitive, including questions about your HIV or use of substances. If you agree to participate, you can decide to skip questions or refuse to answer any questions that you do not want to answer. If you feel you need it, study staff can provide you with basic counseling and information on where and how to access mental and emotional support.

Taking a blood sample for testing has a small risk of pain, bleeding, bruising, and infection. To keep this risk low, all study staff have been trained on how to safely take a blood sample and we will take the smallest of amount of blood necessary to check for cardio-metabolic condition(s) and make sure your HIV is being managed properly. If we see that you had a test done recently at the clinic, like for your viral load, we may not repeat it for the study. The risks involved with giving a blood sample are the same as those you would find when going to the clinic for a regular check-up or to get care for a health problem.

If you choose not to be in the study, what other treatment options do you have?
You do not have to be in this study to receive treatment. If you do not want to participate, you will receive the standard care and treatment for your HIV and any NCDs from the clinic or hospital.

What if we learn about new findings or information during the study?
You will be given any new information learned during the study that might affect your willingness to continue your participation.

How will your privacy be protected?
Every effort will be made to keep your personal information confidential. We will train and supervise all study staff on how to protect your study information. Your study information will only be identified by a secret code to protect your privacy. We will also protect the confidentiality of your study information to the maximum extent possible by handling and storing it securely at all times. We will not share confidential study information, including health or locator information, with anyone outside the study team and your healthcare provider(s), and only with your permission and for the purposes of supporting your routine care and treatment. Study staff will take every precaution to make sure *no* confidential study information is shared with your friends, relatives, or community members. When the results of the study are shared or published, we will not use your name or identify you personally. We may use de-identified data from this study in future research or share it with the Research Coordinating Center overseeing this study without additional consent.

Your records may be reviewed by representatives of the University of Zambia Biomedical Research Ethics Committee, the University of North Carolina IRB, the study sponsor (U.S. National Institutes of Health, NIH) or representative (to include study monitors or the sponsor's Research Coordinating Center at Washington University in St. Louis, USA), or the Zambian Ministry of Health through the National Health Research Authority. However, this information will be kept confidential and will only be used to ensure that the study is being conducted properly, that participant safety is being maintained, and that your records are being stored appropriately.

<u>What if you want to stop before your part in the study is complete?</u>
Your participation in this study is completely voluntary. There is no penalty if you do not join the study or do not complete the study. Even if you agree to join the study, you may choose not to answer questions asked of you or to stop any study procedure at any time. You may withdraw from the study for any reason and at any time. If you choose to withdraw, you may notify the study team by contacting the study phone. If you withdraw from the study, we will not collect any further information from you for study purposes. Any information collected from you before your withdrawal may be discarded if you wish. Your decision to withdraw from the study will not affect your access to health services or your relationship with the people providing you health services.

<u>Will you receive anything for being in this study?</u>
You will be given 100 kwacha at the end of each study visit to pay for the costs of transport to the clinic.

<u>Will it cost you anything to be in this study?</u>
It will not cost you anything to participate in this study.

<u>Who is sponsoring this study?</u>
This research is funded by the Centre for Infectious Disease Research in Zambia (CIDRZ), and the National Institutes of Health (NIH) through the National Heart, Lung, and Blood Institute and Fogarty International Center. This means that the research team is being paid by the sponsor for doing the study. Dr. Michael Herce, one of the principal investigators on this study, participates in unpaid activities which are not part of this study for the Centre for Infectious Disease Research in Zambia (CIDRZ). These activities may include consulting, service on committees or boards, giving speeches, or writing reports.

If you would like more information, please ask the researchers listed in the first page of this form.

<u>What if you have questions about this study?</u>
You have the right to ask and have answered, any questions you may have about this study. If you have questions, complaints, concerns, or if a study-related injury occurs, please contact:

Dr. Wilbroad Mutale
Centre for Infectious Disease Research in Zambia
Plot # 34620, Corner of Lukasu and Danny Pule Roads,
Mass Media, PO Box 34681 Lusaka, ZAMBIA
Tel: +2600967780284
Email: Wilbroad.Mutale@cidrz.org

What if you have questions about your rights as a research participant?

All research on human volunteers is reviewed by a committee that works to protect your rights and welfare. If you have questions about your rights as a study participant, or problems or concerns about how you are being treated in this study, you may contact:

The Chairperson
University of Zambia Biomedical Research Ethics Committee
Ridgeway Campus, Nationalist Road, Lusaka
Landline Telephone: 0211-256-067
Email: unzarec@unza.zm

Informed consent form signature page

Title of study: Z32201- Effects of a package of evidence-based interventions and implementation strategies based on WHO PEN for people living with HIV and cardio-metabolic conditions in Lusaka, Zambia: A type II hybrid effectiveness-implementation stepped wedge trial

Consent form version date: Version 1.1, dated 14 February 2023

Principal Investigators: Dr. Wilbroad Mutale, UNZA & CIDRZ, and Dr. Michael Herce, CIDRZ & UNC

Participant's Agreement

If you agree to join this study, you will need to sign or make your mark below. Before you sign or make your mark on this consent form, make sure of the following:

- I have read this consent form, or someone has read it to me.
- I understand what the study is about and what will happen to me if I join. I understand what the possible risks and benefits are.
- I have had my questions answered and know that I can ask more.
- I agree to join this study.
- I will not be giving up any of my rights by signing this consent form.

____ _______________
Signature/thumbprint of research participant Date

____
Printed name of the research participant

____ _______________
Signature of research team member obtaining consent Date

____
Printed name of research team member obtaining consent

____ _______________
*Signature of witness Date

____
*Printed Name of witness

***Note: Witness name, signature and date are required on this consent form only when the consenting volunteer is not able to read and/or is illiterate**

FOR OFFICIAL USE ONLY

Name……………………………………………………….……

Signature of Principal Investigator ………………………………

Date (DD/MM/YY)…………………………………………….…...
